# A Histone Acetylation Modulator Gene Signature for Classification and Prognosis of Breast Cancer

**DOI:** 10.3390/curroncol28010091

**Published:** 2021-02-17

**Authors:** Mengping Long, Wei Hou, Yiqiang Liu, Taobo Hu

**Affiliations:** 1 Department of Pathology, Peking University Cancer Hospital, Beijing 100142, China; mplong@connect.ust.hk (M.L.); houwei112030@163.com (W.H.); victor.liu@163.com (Y.L.); 2 Department of Breast Surgery, Peking University People’s Hospital, Beijing 100044, China

**Keywords:** histone acetylation, breast cancer, gene signature, prognosis, intrinsic subtype

## Abstract

Regulators of histone acetylation are promising epigenetic targets for therapy in breast cancer. In this study, we comprehensively analyzed the expression of histone acetylation modulator genes in breast cancer using TCGA data sources. A gene signature composed of eight histone acetylation modulators (HAMs) was found to be effective for the classification and prognosis of breast cancers, especially in the HER2-enriched and basal-like molecular subtypes. The eight genes consist of two histone acetylation writers (*GTF3C4* and *CLOCK*), two erasers (*HDAC2* and *SIRT7*) and four readers (*BRD4*, *BRD7*, *SP100,* and *BRWD3*). Both histone acetylation writer genes and eraser genes were found to be differentially expressed between the two groups indicating a close relationship exists between overall histone acetylation level and prognosis of breast cancer in HER2-enriched and basal-like breast cancer.

## 1. Introduction

Breast cancer has overtaken lung cancer as the most commonly diagnosed cancer globally in 2020 [1]. Breast cancer is widely accepted as a highly heterogeneous disease. The current approach to classifying breast cancer into clinical subtypes is based on the immunohistochemistry (IHC) results of estrogen receptor, progesterone receptor, human epidermal growth factor receptor 2, and proliferation marker Ki67. However, this IHC based clinical subtyping system is not ideal, and gene expression profiling (intrinsic subtyping) reveals a deeper appreciation for the disease heterogeneity [2]. In 2000, Perou *et al*. developed the PAM50 intrinsic subtypes of breast cancer based on a set of 50 genes [3]. Since then, various other tests based on gene expression quantification have been developed to provide molecular stratification of breast cancer [4].

Previous pan-cancer studies have revealed that besides genomic alteration, epigenetic changes were common and played an essential role in various cancer types including breast cancer [5,6]. Epigenetic changes generally involve DNA methylation and histone modification which can regulate chromatin structure. DNA methylation has been comprehensively analyzed in breast cancer together with the transcriptomic data and two characterized DNA methylation features named Epi-LumB and Epi-Basal have been identified to indicate poor prognosis [7]. However, studies involving histone modification, particularly histone acetylation are limited in breast cancer. Histone acetylation was a dynamic and reversible process controlled by histone acetylation modulator (HAM) genes including the writers, the erasers and the readers. The acetyl-group was added to the lysine residue of either H3 or H4 by a group of histone acetyltransferases (HATs) which was the writer while the acetyl-group can be removed by specific histone deacetylase (HDAC) which was referred to as the eraser. Additionally, there are proteins called histone acetylation readers that could recognize acetylated histones and recruit transcription machinery [8]. The bromodomain and extra-terminal domain (BET) family was one of the readers which can bind specifically to acetylated H3/H4 and recruit downstream effectors to activate transcription [9]. Histone acetylation is closely related to transcription activation either by directly making the nucleosomes structurally loose and easy for RNA polymerase to go through or by acting as a marker to recruit transcription machinery [10].

Previous studies found that the overall H4 acetylation level was reduced from normal breast epithelium to paired breast cancer tissue [11] and high histone acetylation level was detected in luminal-type of breast cancer, associated with a good prognosis [12]. Moreover, both HDAC inhibitors and BET inhibitors showed promising therapeutic efficacy for breast cancer patients [13,14]. The histone acetylation modulators often work in a network where one acetylation modulator gene can modify multiple lysine sites and each lysine site can be modified by multiple modulators [15]. Moreover, the balance between histone acetylation writer and eraser also contributes to the overall acetylation level. However, most current studies are focused either on a single acetylation site or the acetylation status of a specific target gene. Studies involving a whole set of HAM genes in breast cancer are limited. In this study, we comprehensively analyzed the expression of 73 histone acetylation modulator genes in breast cancer using TCGA data sources. A gene signature composed of eight HAMs was found to be effective for the classification and prognosis prediction of breast cancer.

## 2. Results

### 2.1. A Histone Acetylation Modulator Gene Signature Could Classify Breast Cancers into Two Groups

**Figure 1:**
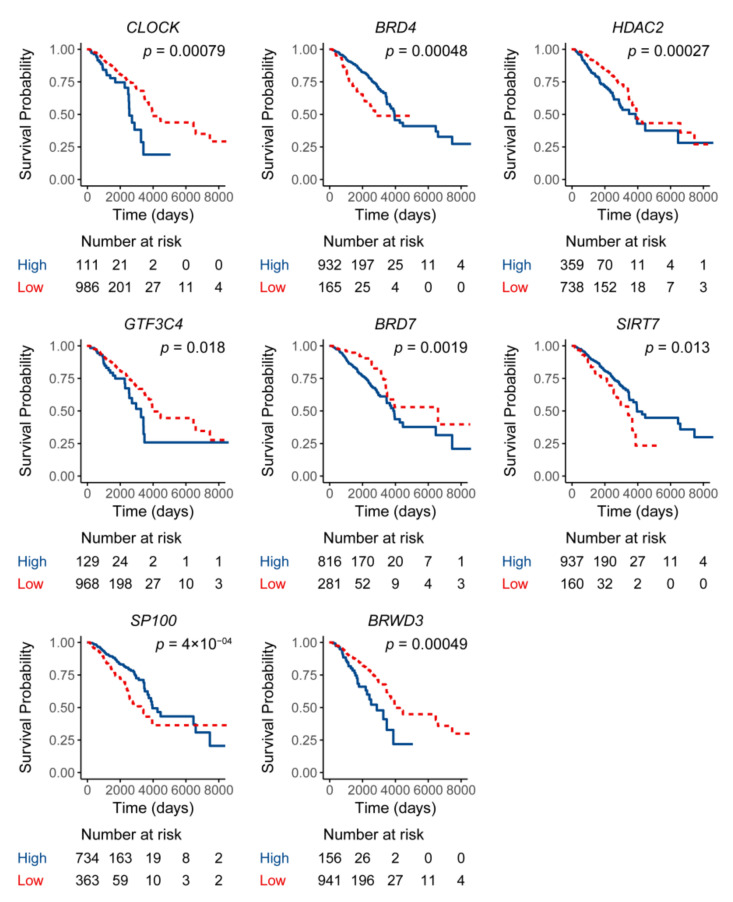
Survival analysis of the eight featured genes. Survival analyses were conducted by Kaplan–Meier method according to the expression level of each specific gene. The optimum cutpoint for distinguishing low and high expression group was determined with the “surv_cutpoint” function.

**Figure 2:**
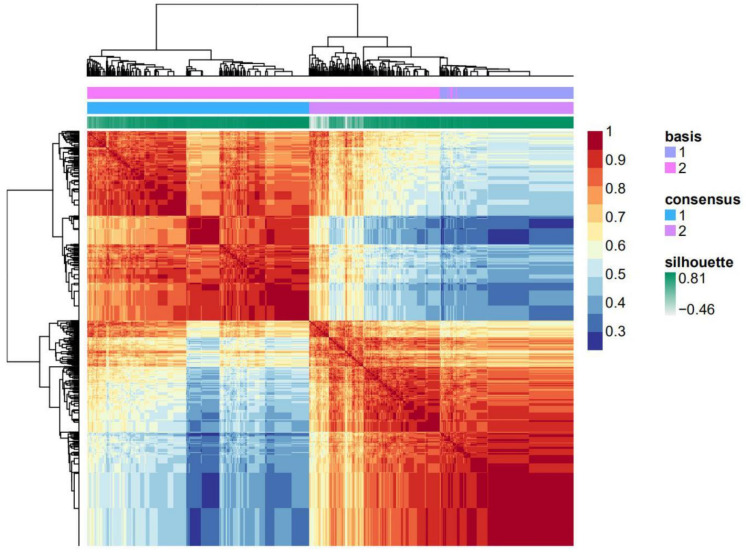
The heatmap of correlation coefficient is clustered by non-negative matrix factorization (NMF). The final clustering generated two groups. One is the rectangle in the upper-left and the other is the rectangle in the lower-right which were named as HAM1 and HAM2, respectively. Each point in the heatmap represents the correlation coefficient between two samples, as displayed by color scale. Three annotation tracks that contributed to the clustering were displayed above the heatmap, including basis components, consensus and silhouette width. Except for the consensus which represents the consistency in the 30 times running of NMF clustering, both the ‘basis component’ and ‘silhouette’ tracks are analyzed in more detail in the following figures.

**Figure 3:**
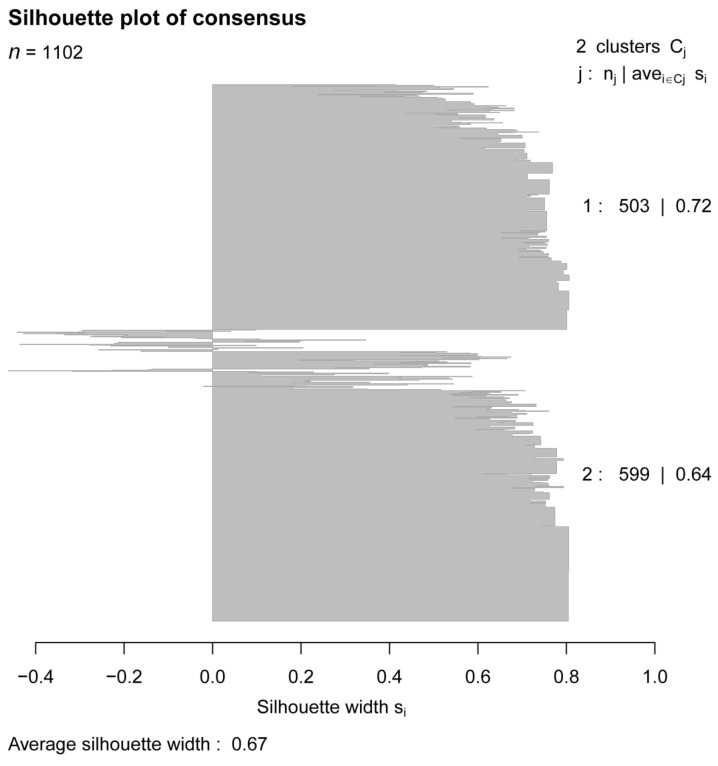
The silhouette width analysis of the clustering using HAM signature. The value of silhouette width represents the cluster validity where a higher average silhouette width indicates higher sample tightness and better cluster separation. Silhouette width for each sample was calculated and displayed by the plot with the average silhouette width for each cluster and total patients presented.

A total of 73 histone acetylation modulator genes were studied as in previous literature [16]. Out of the 73 histone acetylation modulator genes, eight of them were found to be related to breast cancer prognosis in the process of feature selection. The feature selecting process was based on the Cox regression model by the function “FSbyCOX” in the package CancerSubtypes [17]. The survival analyses were performed for the eight genes separately by comparing the overall survival of the high-expression and low-expression groups. The expression of the eight genes were all correlated with the overall survival of breast cancer although in two different trends. Specifically, the high expression of *BRD4*, *SIRT7* and *SP100* were correlated with good prognosis while high expression of the other five genes indicated a poor prognosis (Figure 1). Among the eight genes, there are two histone acetylation writers which are *GTF3C4* and *CLOCK*, two erasers which are *HDAC2* and *SIRT7* as well as four readers which are *BRD4*, *BRD7*, *SP100,* and *BRWD3*. A panel composed of the eight genes was used as a gene signature to further classify breast cancer and was briefed as HAM signature. Transcriptomic data and clinical follow-up of the 1102 breast cancer patients from the TCGA breast cancer cohort were used for the classification. Non-negative matrix factorization (NMF) clustering was used to classify them into two groups named HAM1 and HAM2 (Figure 2). NMF is an efficient unsupervised machine-learning algorithm to identify distinct molecular patterns and molecular classification with high-throughput data. The classification result for each TCGA sample was listed in Table S1. Cluster validity was represented by the average silhouette width, where a higher average silhouette width indicates higher sample tightness and better cluster separation (Figure 3). The average silhouette width for the clustering reached a value of 0.67 with the silhouette width for HAM1 and HAM2 reaching 0.72 and 0.64, respectively. The mRNA expression level of the eight genes in normal breast tissue and breast cancers including four intrinsic subtypes were analyzed in Figure S1. Various patterns of expression were noticed for the eight genes which suggest that the HAM groups could be very different from the PAM50 intrinsic subtypes.

### 2.2. Classification Using Histone Acetylation Modulator Genes Distinguished Two Prognosis Groups in HER2-Enriched and Basal-Like Intrinsic Subtypes

**Figure 4:**
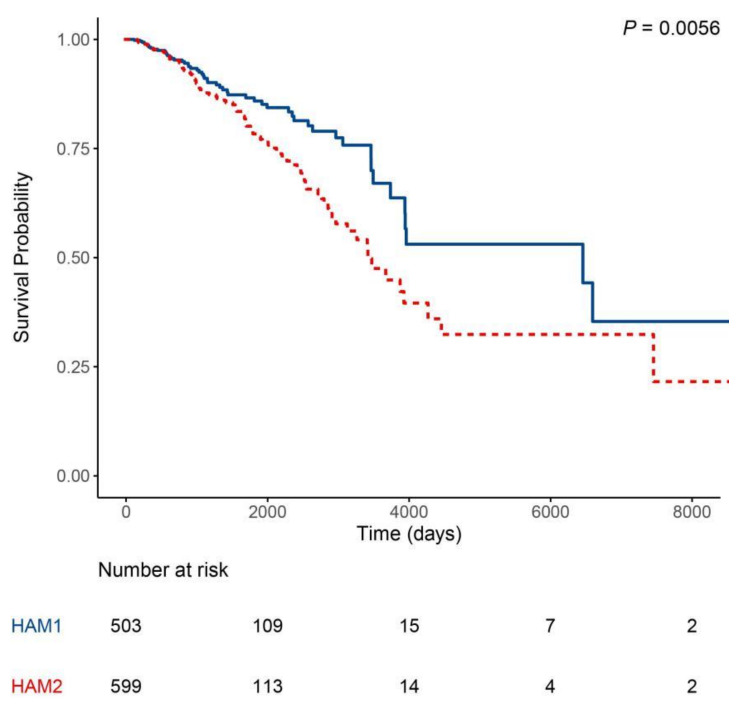
Kaplan–Meier estimate of the overall survival for HAM1 and HAM2 groups of patients.

**Figure 5:**
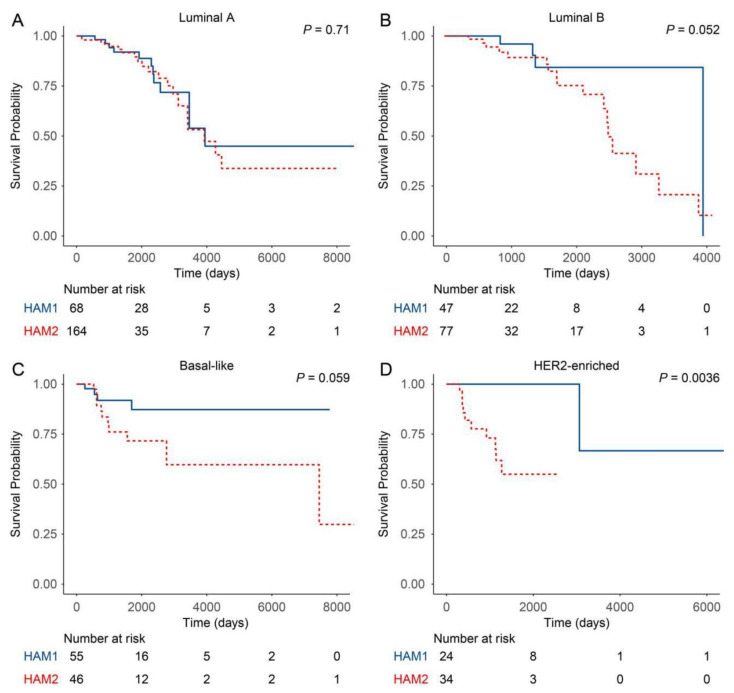
Kaplan–Meier estimate of the overall survival for four intrinsic subtypes between the HAM1 and HAM2 groups.

To further analyze the characteristics of HAM1 and HAM2 groups, the distribution of four intrinsic subtypes in the two groups were calculated and displayed in Table 1. Compared with HAM2, the HAM1 group was more enriched in basal-like and HER2-enriched subtypes. In order to identify the prognostic value for the clustering, survival analysis was conducted comparing the overall survival between HAM1 and HAM2 groups. It was found that the HAM1 group has a better prognosis than the HAM2 group indicating a clinical significance and prognostic value for this classification (Figure 4). Further analysis revealed that it was the HER2-enriched and basal-like subtypes in HAM1 and HAM2 that contributed to the survival difference while Luminal A and B subtypes of the two groups showed no or minor survival differences (Figure 5). These results indicate that in the HER2-enriched and basal-like molecular subtypes the differential expression of the HAM signature indicates a different prognosis. Although whether there is a causal effect between the expression of HAM signature and survival remains elusive, it suggested that the HAM classification can be used as a further stratification of the PAM50 subtypes. Additionally, differentially expressed genes (DEGs) between the two groups were analyzed and listed in Table S2. The volcano plot also showed that genes in HAM1 and HAM2 groups have different expression patterns (Figure S2).

### 2.3. The Eight Featured Genes Belonged to Two Basis Components with a Different Expression Pattern

**Figure 6:**
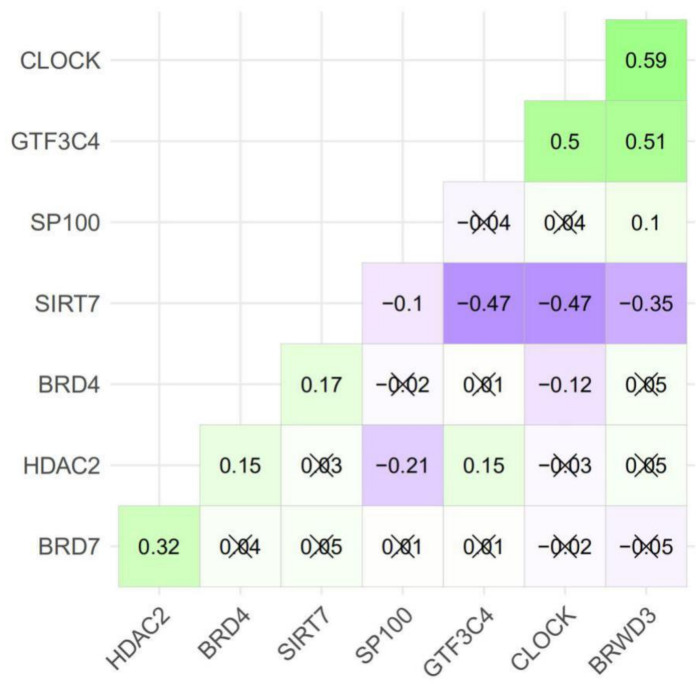
Gene expression correlation plot of the HAM signature genes. Numbers in each square represents the correlation efficiency between the two genes of the specific row and column. Squares with a “×” mark are those insignificantly correlated gene pairs defined by a *p*-value larger than 0.05.

**Figure 7:**
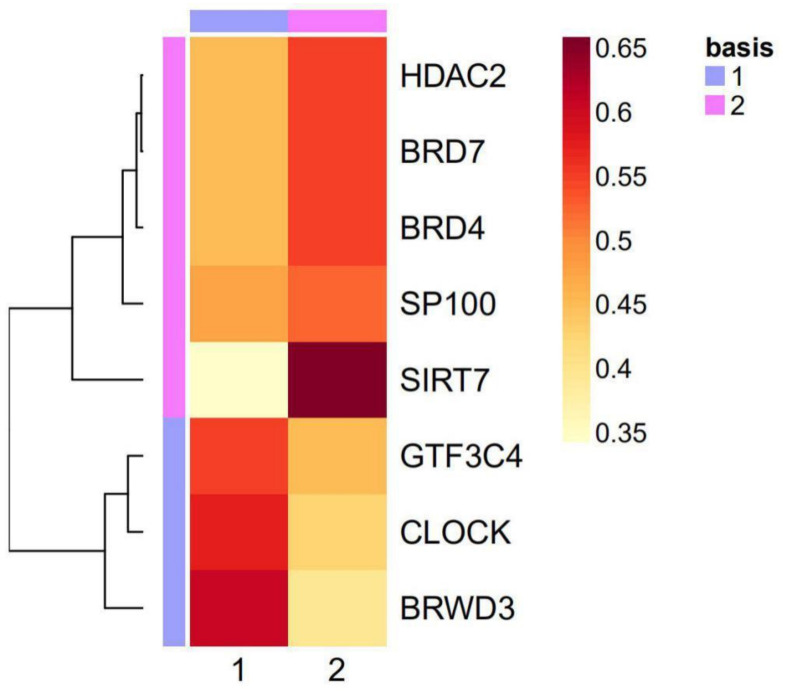
Two major basis components analysis for the NMF clustering. The HAM signature genes can be divided into two basis components with *GTF3C4*, *CLOCK*, and *BRWD3* being one of the components and the other five genes being the other.

**Figure 8:**
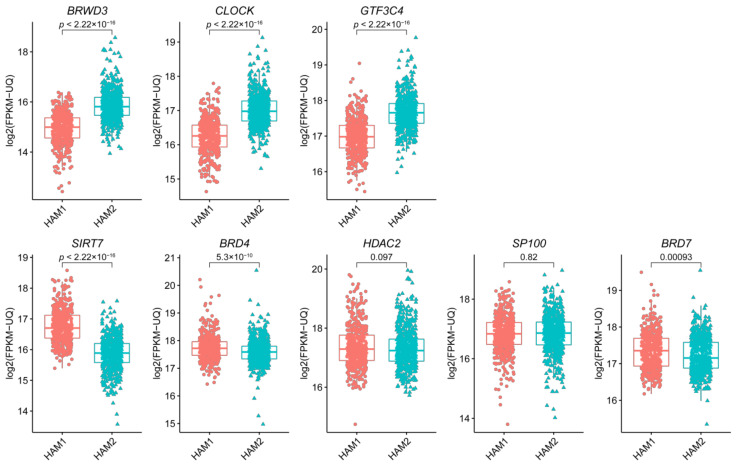
Expression of the eight HAM signature genes in HAM1 and HAM2 groups of patients. All of the eight HAM signature genes, except for *HDAC* and *SP100*, showed significant differential gene expression between HAM1 and HAM2 groups. Genes in the upper row including *BRWD3*, *CLOCK* and *GTF3C4* have higher expression in HAM2 group while those in the lower row including *SIRT7*, *BRD4* and *BRD7* are expressed more in HAM1 group.

Basis component analysis of the NMF clustering revealed that there were two basic components in the eight featured genes with *CLOCK*, *GTF3C4* and *BRWD3* being Basis 1 and the others being Basis 2 (Figure 6 and Figure 7). The expression of genes in Basis 1, *CLOCK*, *GTF3C4* and *BRWD3*, were in a highly positive correlation with each other and correlation among genes in Basis 2 were slightly weaker (Figure 6). Worth to note, all of the writers were in Basis 1 while all erasers were in Basis 2. Additionally, genes in Basis 1 showed a higher expression in HAM2 and genes in Basis 2 expressed more in HAM1, which were all statistically significant except for *HDAC2* and *SP100* (Figure 8).

## 3. Discussion

Histone acetylation modulator genes have been shown to play an essential role in the epigenetic control of breast cancer while its specific role in breast cancer was unequivocal. In this study, a gene signature composed of eight histone acetylation modulator genes was identified which can be used to classify breast cancers into HAM1 and HAM2 groups with their expression value. Significantly, the overall survival of the HER2-enriched and basal-like molecular subtypes between these two groups was different, with HAM1 group showing a much better prognosis. It suggested a clinical significance and prognostic value for the HAM group classification. Specifically, the HAM gene signature can be measured along with the PAM50 as a further stratification. In cases classified as HER2-enriched and basal-like subtypes by PAM50, further classification into HAM1 group would suggest a good prognosis while the classification result of HAM2 indicates a poor prognosis. Indeed, numerous studies have shown that each intrinsic subtype identified by PAM50 was still heterogeneous and a further stratification particularly with a prognostic significance was necessary [18,19,20].

The specific roles of the eight signature genes in breast cancer have been studied before although in different depths. For the two writers, *CLOCK* was reported to have a regulatory role in breast cancer tumorigenesis [21,22], while no specific studies have reported the role of *GTF3C4* in breast cancer. For the two erasers, overexpression of *HDAC2* had a strong effect on breast cancer prognosis [23,24,25] while the effect of *SIRT7* seems to be controversial [26,27,28]. Moreover, the writer genes were found to be more highly expressed in HAM2 than in HAM1 while the erasers showed the opposite trend. However, since the HAM gene signatures comprise only 8 of the 73 HAM genes, the overall histone acetylation level cannot be directly speculated by the expression of the signature genes. Instead, the acetylation status in HAM1 and HAM2 groups should be checked in the breast cancer sample. The function and the correlation of these signature genes should be further explored.

## 4. Materials and Methods

### 4.1. Data Collection and Processing

Data acquisition and analysis were conducted using R software (https://www.r-project.org/, version 4.0.3) unless otherwise mentioned. RNA-seq and clinical data were downloaded from the TCGA dataset [29] using the TCGAbioloinks R/Bioconductor package (version 2.18.0) [30]. Generally, we used TCGAbiolinks to download 1102 breast cancer samples with Illumina HiSeq RNASeqV2 data.

The Fragments Per Kilobase of transcript per Million fragments mapped (FPKM) is the most commonly used normalization method for RNA transcript reads. Upper-quartile normalized FPKM (FPKM-UQ) uses Upper-quartile gene counts rather than total gene counts for normalization and is believed to have better accuracy in gene differential expression identification [31]. In this study, FPKM-UQ RNA-seq data were downloaded and prepared using the GDCquery, GDCdownload, and GDCprepare functions.

### 4.2. Identification and Verification of Featured Genes

For the 73 histone acetylation modulator genes, those significantly associated with a prognostic value were selected using the function “FSbyCOX” in the package CancerSubtypes (version 1.16.0) [17]. The “FSbyCOX” function selected featured genes by the COX regression model. Eight genes were found to have a significant prognostic value while the other 65 genes have no prognostic value in breast cancer. Further verification of the selected genes was performed by analyzing the association between their expression and overall survival. The optimum cutpoint regarding low and high expression threshold was determined with the “surv_cutpoint” function in the “survminer” package in R. Survival analyses were performed for each of the eight genes by comparing the overall survival of the high-expression and low-expression group.

### 4.3. Nonnegative Matrix Factorization Clustering

NMF is a clustering method widely used for cancer molecular subtyping using gene expression data [32,33]. Standard ‘brunet’ for 30 iterations was selected by NMF, using the R package ‘NMF’ [34]. In the clustering, the correlation coefficient of each two random samples was calculated using the expression value of the eight feature genes. All of the correlation values can then be plotted in an 1102 × 1102 matrix with each row and column representing one sample in the same order. NMF algorithm was used for the clustering by setting the number of the components to 2 (κ = 2). Silhouette widths were generated from the NMF consensus membership matrix to represent the cluster validity.

### 4.4. Survival Analysis

Survival analyses were performed using the ‘survival’ (version 2.41) package [35]. The Kaplan–Meier method was used to estimate the survival outcomes of all patients by different categories; groups were compared using the log-rank statistic [36]. *p*-values were calculated as two-sided, with statistical significance declared for *p* less than 0.05.

### 4.5. Analysis of Differentially Expressed Genes between the HAM1 and HAM2 Groups

The log2-transformed FPKM-UQ data were analyzed using limma (Version 3.46.0) package [37] functions lmFit, eBayes, and topTable to identify DEGs between HAM1 and HAM2 groups of patients. Student’s *t*-test was utilized to calculate the *p* values of genes. Genes with *p* < 0.05 were considered as DEGs.

## Figures and Tables

**Table 1:** Distribution of four molecular intrinsic subtypes in HAM1 and HAM2 groups.

HAM Groups	Luminal A	Luminal B	Basal-Like	HER2-Enriched
HAM1	68 (35.1%)	47 (24.2%)	55 (28.3%)	24 (12.4%)
HAM2	164 (50.9%)	78 (24.2%)	46 (14.3%)	34 (10.6%)

A Chi-squared test was performed to compare the distribution of four intrinsic subtypes between the two groups with the *p*-value found to be 0.00023.

## Data Availability

The data presented in this study are available in the article and supplementary material, and are also available on request from the corresponding author.

## Supplementary Materials

The following are available online at https://www.mdpi.com/1718-7729/28/1/91/s1, Figure S1: Expression of the eight HAM signature genes in normal breast tissues and breast cancers with four PAM50 intrinsic molecular subtypes, Figure S2: Volcano plot of differentially expressed genes between HAM1 and HAM2 groups, Table S1: HAM group classification result for each TCGA sample, Table S2: List of differentially expressed genes between HAM1 and HAM2.
