# Supplementary material for: A Histone Acetylation Modulator Gene Signature for Classification and Prognosis of Breast Cancer
Source: Curr Oncol. 2021 Feb 17;28(1):928–39. doi: 10.3390/curroncol28010091 (PMC7985767; doi:10.3390/curroncol28010091)
Supplement: Supplementary file 1 [file curroncol-28-00091-s001.zip › Table S2.docx]

| **Name** | **Id** | **logFC** | **AveExpr** | **t** | **P.Value** | **adj.P.Val** | **B** |
| --- | --- | --- | --- | --- | --- | --- | --- |
| ZBED6 | ENSG00000257315 | 2.510 | 2.605 | 23.253 | 0.000 | 0.000 | 211.868 |
| PWAR5 | ENSG00000279192 | 2.475 | -0.702 | 17.871 | 0.000 | 0.000 | 132.358 |
| AC092881.1 | ENSG00000279530 | 2.926 | -2.654 | 17.617 | 0.000 | 0.000 | 128.862 |
| ZDHHC20P4 | ENSG00000232380 | 3.081 | -2.753 | 16.953 | 0.000 | 0.000 | 119.851 |
| AC018752.1 | ENSG00000250015 | 3.332 | 0.385 | 16.699 | 0.000 | 0.000 | 116.462 |
| AC087286.2 | ENSG00000259367 | 3.048 | -0.685 | 16.434 | 0.000 | 0.000 | 112.951 |
| OTUD4P1 | ENSG00000118976 | 2.496 | -4.528 | 16.303 | 0.000 | 0.000 | 111.237 |
| ERVW-1 | ENSG00000242950 | 2.344 | -4.314 | 16.016 | 0.000 | 0.000 | 107.493 |
| AC007673.1 | ENSG00000267311 | 3.584 | -5.200 | 15.253 | 0.000 | 0.000 | 97.750 |
| AL590326.2 | ENSG00000279140 | -3.208 | -0.815 | -15.215 | 0.000 | 0.000 | 97.270 |
| TAS2R3 | ENSG00000127362 | 3.449 | -4.912 | 15.110 | 0.000 | 0.000 | 95.958 |
| AP000811.1 | ENSG00000270510 | 2.590 | -1.211 | 14.976 | 0.000 | 0.000 | 94.287 |
| RN7SKP74 | ENSG00000199719 | 3.691 | -2.402 | 14.780 | 0.000 | 0.000 | 91.874 |
| AC253536.3 | ENSG00000232545 | 2.444 | 0.261 | 14.675 | 0.000 | 0.000 | 90.584 |
| Y_RNA | ENSG00000201217 | 4.288 | -2.026 | 14.510 | 0.000 | 0.000 | 88.568 |
| AP002495.1 | ENSG00000225805 | 2.980 | -0.303 | 14.487 | 0.000 | 0.000 | 88.285 |
| AC090181.3 | ENSG00000278991 | 2.265 | -2.171 | 14.453 | 0.000 | 0.000 | 87.874 |
| MIR421 | ENSG00000202566 | 4.080 | -1.678 | 14.432 | 0.000 | 0.000 | 87.617 |
| AC094019.1 | ENSG00000236732 | 3.153 | -1.417 | 14.207 | 0.000 | 0.000 | 84.908 |
| HMGN1P8 | ENSG00000241120 | 3.282 | -1.926 | 13.974 | 0.000 | 0.000 | 82.135 |
| AC022395.1 | ENSG00000231104 | 2.626 | -4.551 | 13.864 | 0.000 | 0.000 | 80.832 |
| AC108693.2 | ENSG00000242659 | 3.286 | -2.493 | 13.813 | 0.000 | 0.000 | 80.232 |
| NADK2-AS1 | ENSG00000245711 | 2.393 | -0.847 | 13.719 | 0.000 | 0.000 | 79.136 |
| AC100774.1 | ENSG00000261529 | 2.685 | -4.205 | 13.697 | 0.000 | 0.000 | 78.874 |
| MIR5692C2 | ENSG00000266668 | 4.314 | -6.058 | 13.467 | 0.000 | 0.000 | 76.205 |
| AC064801.2 | ENSG00000279236 | 2.641 | -3.486 | 13.443 | 0.000 | 0.000 | 75.924 |
| RNU6-531P | ENSG00000252503 | 2.762 | 1.172 | 13.420 | 0.000 | 0.000 | 75.661 |
| AL021068.2 | ENSG00000234604 | 3.462 | 0.897 | 13.396 | 0.000 | 0.000 | 75.386 |
| LINC02211 | ENSG00000245662 | 3.106 | -5.311 | 13.210 | 0.000 | 0.000 | 73.258 |
| AC093012.1 | ENSG00000257896 | 2.307 | -2.524 | 13.197 | 0.000 | 0.000 | 73.112 |
| HMGB3P14 | ENSG00000231845 | 3.201 | -4.618 | 13.113 | 0.000 | 0.000 | 72.160 |
| VEZF1P1 | ENSG00000236229 | 2.654 | -7.070 | 13.044 | 0.000 | 0.000 | 71.376 |
| AL365356.1 | ENSG00000226647 | 2.500 | -3.247 | 13.043 | 0.000 | 0.000 | 71.369 |
| AC124312.2 | ENSG00000257647 | 2.916 | -0.845 | 12.975 | 0.000 | 0.000 | 70.599 |
| RNA5SP317 | ENSG00000222108 | 3.336 | -0.747 | 12.970 | 0.000 | 0.000 | 70.541 |
| AC093849.1 | ENSG00000261646 | 2.619 | -5.451 | 12.885 | 0.000 | 0.000 | 69.589 |
| RPL36AP15 | ENSG00000235369 | 3.004 | -2.248 | 12.851 | 0.000 | 0.000 | 69.214 |
| AC124312.4 | ENSG00000270704 | 3.484 | -2.914 | 12.780 | 0.000 | 0.000 | 68.425 |
| BNIP3P26 | ENSG00000268705 | 2.996 | -3.386 | 12.761 | 0.000 | 0.000 | 68.219 |
| NUDT19P5 | ENSG00000251073 | 3.397 | -4.102 | 12.723 | 0.000 | 0.000 | 67.787 |
| Y_RNA | ENSG00000212306 | 3.994 | -5.867 | 12.695 | 0.000 | 0.000 | 67.484 |
| RNU6-652P | ENSG00000202358 | 3.590 | -1.317 | 12.667 | 0.000 | 0.000 | 67.171 |
| RN7SL431P | ENSG00000264384 | 2.739 | -1.245 | 12.620 | 0.000 | 0.000 | 66.660 |
| ARL4AP4 | ENSG00000231395 | 2.807 | -2.412 | 12.607 | 0.000 | 0.000 | 66.510 |
| RHOT1P3 | ENSG00000242756 | 3.345 | -3.830 | 12.589 | 0.000 | 0.000 | 66.317 |
| AC108693.1 | ENSG00000239280 | 3.350 | -5.263 | 12.560 | 0.000 | 0.000 | 65.997 |
| AL445435.1 | ENSG00000270585 | 2.848 | -3.430 | 12.505 | 0.000 | 0.000 | 65.395 |
| FAM47E-STBD1 | ENSG00000272414 | 2.130 | -4.187 | 12.455 | 0.000 | 0.000 | 64.854 |
| AC012065.2 | ENSG00000233416 | 3.115 | -3.496 | 12.439 | 0.000 | 0.000 | 64.673 |
| AC008507.2 | ENSG00000267006 | 2.623 | -4.434 | 12.418 | 0.000 | 0.000 | 64.441 |
| AC068790.1 | ENSG00000256827 | 3.190 | -3.721 | 12.326 | 0.000 | 0.000 | 63.447 |
| PHACTR2P1 | ENSG00000270300 | 2.375 | -3.822 | 12.200 | 0.000 | 0.000 | 62.092 |
| PCCA-AS1 | ENSG00000234650 | 2.856 | -4.625 | 12.180 | 0.000 | 0.000 | 61.874 |
| OPA1-AS1 | ENSG00000224855 | 2.522 | -2.460 | 12.167 | 0.000 | 0.000 | 61.740 |
| AC002400.1 | ENSG00000261528 | 2.846 | -3.752 | 12.122 | 0.000 | 0.000 | 61.260 |
| SPATA46 | ENSG00000171722 | 2.329 | -1.425 | 12.117 | 0.000 | 0.000 | 61.199 |
| RNU7-84P | ENSG00000251892 | 4.060 | -5.856 | 12.113 | 0.000 | 0.000 | 61.159 |
| RNA5SP187 | ENSG00000207129 | 3.319 | -0.991 | 12.087 | 0.000 | 0.000 | 60.881 |
| AL683842.1 | ENSG00000232334 | 2.837 | -3.078 | 12.055 | 0.000 | 0.000 | 60.542 |
| HSPD1P11 | ENSG00000251348 | 2.480 | -3.581 | 12.034 | 0.000 | 0.000 | 60.326 |
| AF127577.6 | ENSG00000279390 | 2.498 | -4.508 | 12.031 | 0.000 | 0.000 | 60.286 |
| AL590282.1 | ENSG00000274024 | 2.170 | -2.504 | 12.009 | 0.000 | 0.000 | 60.059 |
| AC016590.2 | ENSG00000267682 | 2.059 | 0.034 | 11.988 | 0.000 | 0.000 | 59.834 |
| IL6STP1 | ENSG00000227018 | 2.897 | -5.369 | 11.949 | 0.000 | 0.000 | 59.421 |
| Y_RNA | ENSG00000252652 | 3.814 | -3.636 | 11.925 | 0.000 | 0.000 | 59.167 |
| RNU6-130P | ENSG00000223044 | 3.862 | -4.432 | 11.907 | 0.000 | 0.000 | 58.978 |
| RNU6-638P | ENSG00000252391 | 3.675 | -2.590 | 11.907 | 0.000 | 0.000 | 58.975 |
| Z99129.1 | ENSG00000275339 | 3.043 | -2.446 | 11.894 | 0.000 | 0.000 | 58.843 |
| MED28P3 | ENSG00000227692 | 2.350 | -1.892 | 11.858 | 0.000 | 0.000 | 58.466 |
| RN7SL262P | ENSG00000265033 | 2.968 | -2.365 | 11.818 | 0.000 | 0.000 | 58.049 |
| AL357568.2 | ENSG00000271917 | 2.631 | -5.348 | 11.735 | 0.000 | 0.000 | 57.182 |
| CNOT6LP1 | ENSG00000230183 | 2.021 | -3.073 | 11.728 | 0.000 | 0.000 | 57.107 |
| AC010333.1 | ENSG00000259876 | 2.988 | -5.356 | 11.719 | 0.000 | 0.000 | 57.013 |
| GABPAP | ENSG00000235720 | 2.392 | -5.806 | 11.679 | 0.000 | 0.000 | 56.595 |
| EFCAB14-AS1 | ENSG00000228237 | 2.277 | -2.282 | 11.586 | 0.000 | 0.000 | 55.636 |
| AL445933.2 | ENSG00000230881 | 3.352 | -3.272 | 11.585 | 0.000 | 0.000 | 55.619 |
| LYPLA1P3 | ENSG00000218350 | 2.237 | -2.627 | 11.583 | 0.000 | 0.000 | 55.603 |
| RNU6ATAC16P | ENSG00000221518 | 3.356 | -2.688 | 11.580 | 0.000 | 0.000 | 55.574 |
| RNU6-1016P | ENSG00000252498 | 2.418 | 1.776 | 11.551 | 0.000 | 0.000 | 55.270 |
| ZNF728 | ENSG00000269067 | 2.283 | -3.440 | 11.545 | 0.000 | 0.000 | 55.212 |
| MIR374B | ENSG00000212027 | 3.670 | -3.435 | 11.508 | 0.000 | 0.000 | 54.829 |
| PRDX3P4 | ENSG00000239926 | 2.920 | -5.356 | 11.496 | 0.000 | 0.000 | 54.704 |
| AC005387.1 | ENSG00000268938 | -2.399 | -1.520 | -11.487 | 0.000 | 0.000 | 54.618 |
| Y_RNA | ENSG00000202522 | 3.241 | -0.034 | 11.354 | 0.000 | 0.000 | 53.257 |
| AC089998.3 | ENSG00000257875 | 2.926 | -4.612 | 11.337 | 0.000 | 0.000 | 53.090 |
| AL353748.2 | ENSG00000275017 | 2.743 | -2.683 | 11.333 | 0.000 | 0.000 | 53.053 |
| AC005020.1 | ENSG00000224448 | 2.197 | -2.548 | 11.332 | 0.000 | 0.000 | 53.041 |
| ERI3-IT1 | ENSG00000233602 | 2.266 | -2.291 | 11.268 | 0.000 | 0.000 | 52.395 |
| LINC02278 | ENSG00000251635 | 3.059 | -4.718 | 11.268 | 0.000 | 0.000 | 52.387 |
| AC010333.2 | ENSG00000259899 | 2.764 | -5.538 | 11.250 | 0.000 | 0.000 | 52.210 |
| AC115989.2 | ENSG00000264932 | 2.902 | -4.698 | 11.237 | 0.000 | 0.000 | 52.078 |
| AC016644.1 | ENSG00000235635 | 2.999 | -5.437 | 11.229 | 0.000 | 0.000 | 51.998 |
| Metazoa_SRP | ENSG00000274011 | 2.879 | -2.201 | 11.204 | 0.000 | 0.000 | 51.746 |
| Y_RNA | ENSG00000212205 | 3.749 | -4.527 | 11.203 | 0.000 | 0.000 | 51.741 |
| ZNF322P1 | ENSG00000188801 | 2.388 | -5.232 | 11.179 | 0.000 | 0.000 | 51.498 |
| AC067942.2 | ENSG00000248113 | 2.422 | -6.050 | 11.173 | 0.000 | 0.000 | 51.442 |
| SNORD116-24 | ENSG00000207279 | 3.583 | -2.249 | 11.153 | 0.000 | 0.000 | 51.235 |
| AC073415.1 | ENSG00000227470 | 2.192 | 0.413 | 11.089 | 0.000 | 0.000 | 50.593 |
| AC002064.2 | ENSG00000234459 | 2.717 | -0.823 | 11.070 | 0.000 | 0.000 | 50.411 |
| MIR5581 | ENSG00000263675 | 2.934 | 0.854 | 11.069 | 0.000 | 0.000 | 50.397 |
| HMGB3P4 | ENSG00000228808 | 2.561 | -3.066 | 11.007 | 0.000 | 0.000 | 49.780 |
| AC107021.1 | ENSG00000243415 | 2.545 | -3.584 | 11.001 | 0.000 | 0.000 | 49.726 |
| Z95152.1 | ENSG00000237719 | 2.059 | -1.935 | 10.987 | 0.000 | 0.000 | 49.585 |
| AL353583.1 | ENSG00000279029 | 2.007 | -3.484 | 10.969 | 0.000 | 0.000 | 49.410 |
| AC079416.1 | ENSG00000261056 | 2.000 | -3.659 | 10.961 | 0.000 | 0.000 | 49.330 |
| AC139769.3 | ENSG00000277493 | 2.190 | 0.458 | 10.948 | 0.000 | 0.000 | 49.197 |
| WASF4P | ENSG00000188459 | 2.048 | -4.224 | 10.944 | 0.000 | 0.000 | 49.157 |
| AC131953.1 | ENSG00000250906 | 2.195 | -0.912 | 10.930 | 0.000 | 0.000 | 49.026 |
| MIR4312 | ENSG00000265195 | 3.663 | -3.404 | 10.917 | 0.000 | 0.000 | 48.896 |
| SRPK2P | ENSG00000253431 | 2.366 | -5.509 | 10.873 | 0.000 | 0.000 | 48.464 |
| SLC2A3P2 | ENSG00000185031 | 2.368 | -4.289 | 10.861 | 0.000 | 0.000 | 48.348 |
| RNY4P25 | ENSG00000238711 | 3.615 | -4.090 | 10.841 | 0.000 | 0.000 | 48.148 |
| uc_338 | ENSG00000277948 | 3.117 | -3.527 | 10.807 | 0.000 | 0.000 | 47.815 |
| SNORA9 | ENSG00000252192 | 3.235 | -2.817 | 10.792 | 0.000 | 0.000 | 47.676 |
| AC006518.1 | ENSG00000256651 | 2.553 | -5.150 | 10.782 | 0.000 | 0.000 | 47.580 |
| PFN1P3 | ENSG00000234367 | 2.758 | -3.766 | 10.749 | 0.000 | 0.000 | 47.253 |
| BX322635.1 | ENSG00000270497 | 2.862 | -5.813 | 10.698 | 0.000 | 0.000 | 46.762 |
| RNU6-930P | ENSG00000212240 | 3.308 | -2.759 | 10.682 | 0.000 | 0.000 | 46.607 |
| AF241728.1 | ENSG00000235806 | 2.232 | -4.129 | 10.656 | 0.000 | 0.000 | 46.352 |
| VCAN-AS1 | ENSG00000249835 | 2.475 | -3.793 | 10.637 | 0.000 | 0.000 | 46.174 |
| ERVFRD-3 | ENSG00000264801 | 2.151 | -4.220 | 10.636 | 0.000 | 0.000 | 46.160 |
| AC006946.1 | ENSG00000235328 | 2.852 | -3.882 | 10.634 | 0.000 | 0.000 | 46.139 |
| AC105389.2 | ENSG00000249216 | 2.673 | -2.982 | 10.615 | 0.000 | 0.000 | 45.956 |
| AC104115.2 | ENSG00000253908 | 2.310 | -2.919 | 10.613 | 0.000 | 0.000 | 45.941 |
| AC115085.1 | ENSG00000267203 | 2.892 | -2.020 | 10.607 | 0.000 | 0.000 | 45.886 |
| AL049637.1 | ENSG00000225767 | 2.836 | -5.704 | 10.607 | 0.000 | 0.000 | 45.884 |
| AC011753.4 | ENSG00000237916 | 2.298 | -0.878 | 10.595 | 0.000 | 0.000 | 45.767 |
| AC004975.1 | ENSG00000224629 | 2.625 | -1.345 | 10.583 | 0.000 | 0.000 | 45.655 |
| AC007956.1 | ENSG00000258439 | 2.356 | -3.160 | 10.577 | 0.000 | 0.000 | 45.596 |
| SCDP1 | ENSG00000226549 | 2.507 | -4.818 | 10.567 | 0.000 | 0.000 | 45.502 |
| AC022973.3 | ENSG00000254263 | 2.689 | -3.631 | 10.564 | 0.000 | 0.000 | 45.468 |
| AC007919.1 | ENSG00000224611 | 3.186 | -2.835 | 10.558 | 0.000 | 0.000 | 45.410 |
| ANKRD26P4 | ENSG00000229427 | 2.391 | -4.812 | 10.541 | 0.000 | 0.000 | 45.252 |
| AL050341.1 | ENSG00000231296 | 2.810 | -3.559 | 10.533 | 0.000 | 0.000 | 45.174 |
| AF274573.1 | ENSG00000228728 | 2.366 | -2.490 | 10.525 | 0.000 | 0.000 | 45.097 |
| HNRNPA3P11 | ENSG00000260689 | 2.055 | -3.548 | 10.519 | 0.000 | 0.000 | 45.037 |
| RNA5SP82 | ENSG00000252516 | 2.442 | 1.095 | 10.516 | 0.000 | 0.000 | 45.014 |
| AC005154.5 | ENSG00000264520 | 2.105 | -1.085 | 10.489 | 0.000 | 0.000 | 44.757 |
| AC083798.1 | ENSG00000242531 | 3.003 | -1.966 | 10.449 | 0.000 | 0.000 | 44.376 |
| AC019072.1 | ENSG00000271639 | 2.311 | -7.102 | 10.445 | 0.000 | 0.000 | 44.335 |
| AL162431.4 | ENSG00000270711 | 2.757 | -4.664 | 10.422 | 0.000 | 0.000 | 44.114 |
| SNORD116-4 | ENSG00000275529 | 3.273 | -2.155 | 10.421 | 0.000 | 0.000 | 44.113 |
| AC234778.2 | ENSG00000261212 | 2.857 | -4.878 | 10.410 | 0.000 | 0.000 | 44.002 |
| PDSS1P1 | ENSG00000182347 | 2.439 | -6.173 | 10.386 | 0.000 | 0.000 | 43.780 |
| Y_RNA | ENSG00000199332 | 2.761 | 0.200 | 10.385 | 0.000 | 0.000 | 43.765 |
| AC006441.1 | ENSG00000263466 | 2.211 | -1.694 | 10.349 | 0.000 | 0.000 | 43.431 |
| uc_338 | ENSG00000275227 | 2.956 | -2.574 | 10.343 | 0.000 | 0.000 | 43.371 |
| YWHAQP6 | ENSG00000249986 | 2.778 | -3.864 | 10.338 | 0.000 | 0.000 | 43.328 |
| AL139317.4 | ENSG00000271075 | 2.484 | -3.886 | 10.316 | 0.000 | 0.000 | 43.116 |
| CYCSP52 | ENSG00000235700 | 2.727 | -3.454 | 10.311 | 0.000 | 0.000 | 43.071 |
| AL445187.1 | ENSG00000250068 | 2.130 | -0.251 | 10.307 | 0.000 | 0.000 | 43.036 |
| AC027020.1 | ENSG00000259540 | 2.328 | -2.657 | 10.290 | 0.000 | 0.000 | 42.877 |
| AC134775.1 | ENSG00000255165 | 2.604 | -3.694 | 10.272 | 0.000 | 0.000 | 42.710 |
| TCEAL3-AS1 | ENSG00000224031 | 2.775 | -4.465 | 10.271 | 0.000 | 0.000 | 42.701 |
| Y_RNA | ENSG00000251728 | 3.100 | -1.836 | 10.257 | 0.000 | 0.000 | 42.566 |
| SMAD1-AS2 | ENSG00000250582 | 2.351 | -7.450 | 10.255 | 0.000 | 0.000 | 42.547 |
| AC004066.2 | ENSG00000250522 | 2.184 | -1.329 | 10.229 | 0.000 | 0.000 | 42.301 |
| MIR5692A1 | ENSG00000266318 | 3.270 | -6.868 | 10.222 | 0.000 | 0.000 | 42.238 |
| RNA5SP123 | ENSG00000200114 | 3.256 | -4.068 | 10.219 | 0.000 | 0.000 | 42.212 |
| ALG13-AS1 | ENSG00000229487 | 2.037 | 0.262 | 10.164 | 0.000 | 0.000 | 41.696 |
| TPRKBP2 | ENSG00000260452 | 2.649 | -6.536 | 10.159 | 0.000 | 0.000 | 41.653 |
| AL391840.1 | ENSG00000261970 | 2.729 | -4.300 | 10.132 | 0.000 | 0.000 | 41.400 |
| GCNT1P1 | ENSG00000236474 | 2.270 | -4.737 | 10.131 | 0.000 | 0.000 | 41.397 |
| SCARNA18 | ENSG00000238835 | 3.070 | -6.673 | 10.116 | 0.000 | 0.000 | 41.259 |
| MIR3657 | ENSG00000266370 | 3.217 | -5.317 | 10.109 | 0.000 | 0.000 | 41.191 |
| TGFB3-AS1 | ENSG00000258876 | 2.346 | -3.128 | 10.088 | 0.000 | 0.000 | 40.998 |
| AC133473.1 | ENSG00000236489 | 2.707 | -3.482 | 10.025 | 0.000 | 0.000 | 40.418 |
| RNU6-100P | ENSG00000252414 | 3.015 | -2.436 | 10.021 | 0.000 | 0.000 | 40.384 |
| FCF1P1 | ENSG00000227436 | 2.503 | -4.606 | 9.990 | 0.000 | 0.000 | 40.096 |
| COMMD3-BMI1 | ENSG00000269897 | 2.229 | -5.330 | 9.961 | 0.000 | 0.000 | 39.830 |
| AL158212.4 | ENSG00000279315 | 2.238 | -6.734 | 9.932 | 0.000 | 0.000 | 39.567 |
| RNU6-282P | ENSG00000202227 | 3.080 | -3.040 | 9.931 | 0.000 | 0.000 | 39.562 |
| AC010285.2 | ENSG00000249412 | 2.279 | -3.300 | 9.930 | 0.000 | 0.000 | 39.552 |
| SEC62-AS1 | ENSG00000240373 | 2.116 | -1.591 | 9.910 | 0.000 | 0.000 | 39.367 |
| AC027544.2 | ENSG00000256588 | 2.340 | -3.916 | 9.890 | 0.000 | 0.000 | 39.190 |
| TAF9BP1 | ENSG00000213871 | 2.438 | -5.817 | 9.868 | 0.000 | 0.000 | 38.986 |
| AL450996.1 | ENSG00000225154 | 2.780 | -3.808 | 9.859 | 0.000 | 0.000 | 38.910 |
| Y_RNA | ENSG00000207034 | 3.037 | -2.740 | 9.857 | 0.000 | 0.000 | 38.884 |
| AC022150.3 | ENSG00000269349 | 2.274 | -4.012 | 9.854 | 0.000 | 0.000 | 38.859 |
| AC092042.1 | ENSG00000227245 | 2.403 | -3.535 | 9.852 | 0.000 | 0.000 | 38.843 |
| AC131391.1 | ENSG00000260601 | 2.506 | -7.246 | 9.845 | 0.000 | 0.000 | 38.777 |
| MTND1P8 | ENSG00000263177 | 2.424 | -4.658 | 9.837 | 0.000 | 0.000 | 38.710 |
| MTCO1P28 | ENSG00000262118 | 2.123 | -4.190 | 9.836 | 0.000 | 0.000 | 38.702 |
| AL713922.2 | ENSG00000276662 | 2.136 | -2.671 | 9.828 | 0.000 | 0.000 | 38.624 |
| SPRY4-IT1 | ENSG00000281881 | 2.089 | -2.400 | 9.823 | 0.000 | 0.000 | 38.586 |
| AP005131.4 | ENSG00000267529 | 2.038 | -4.060 | 9.817 | 0.000 | 0.000 | 38.525 |
| AC021171.1 | ENSG00000270528 | 2.448 | -6.299 | 9.813 | 0.000 | 0.000 | 38.493 |
| AL133406.1 | ENSG00000216639 | 2.453 | -5.417 | 9.794 | 0.000 | 0.000 | 38.317 |
| LINC02463 | ENSG00000257683 | 2.570 | -3.500 | 9.775 | 0.000 | 0.000 | 38.151 |
| RNY4P17 | ENSG00000201818 | 3.111 | -6.393 | 9.772 | 0.000 | 0.000 | 38.125 |
| ARHGAP42P2 | ENSG00000231147 | 2.176 | -6.046 | 9.757 | 0.000 | 0.000 | 37.987 |
| AC090607.1 | ENSG00000259322 | 2.021 | -1.901 | 9.747 | 0.000 | 0.000 | 37.897 |
| OR5BK1P | ENSG00000257763 | 2.108 | -3.491 | 9.741 | 0.000 | 0.000 | 37.844 |
| AC005828.6 | ENSG00000271749 | 2.723 | -2.826 | 9.738 | 0.000 | 0.000 | 37.818 |
| AC024267.5 | ENSG00000265908 | 2.269 | -3.085 | 9.737 | 0.000 | 0.000 | 37.811 |
| Y_RNA | ENSG00000200419 | 2.787 | -7.746 | 9.711 | 0.000 | 0.000 | 37.581 |
| SNORA58 | ENSG00000249020 | 3.034 | -5.356 | 9.671 | 0.000 | 0.000 | 37.218 |
| OR52V1P | ENSG00000249633 | 2.325 | -7.073 | 9.666 | 0.000 | 0.000 | 37.178 |
| AC007362.1 | ENSG00000225216 | 2.144 | -5.382 | 9.658 | 0.000 | 0.000 | 37.103 |
| uc_338 | ENSG00000278236 | 3.006 | -4.884 | 9.644 | 0.000 | 0.000 | 36.980 |
| Y_RNA | ENSG00000239040 | 2.353 | -0.004 | 9.613 | 0.000 | 0.000 | 36.706 |
| AC023043.2 | ENSG00000267404 | 2.343 | -4.957 | 9.611 | 0.000 | 0.000 | 36.687 |
| ITGB5-AS1 | ENSG00000244286 | 2.618 | -4.121 | 9.603 | 0.000 | 0.000 | 36.613 |
| AC020910.3 | ENSG00000271049 | 2.118 | -3.908 | 9.572 | 0.000 | 0.000 | 36.344 |
| RNU6-301P | ENSG00000207004 | 3.089 | -5.904 | 9.564 | 0.000 | 0.000 | 36.272 |
| CLIC4P1 | ENSG00000236739 | 2.455 | -5.995 | 9.563 | 0.000 | 0.000 | 36.260 |
| RNU6-125P | ENSG00000207234 | 3.034 | -3.398 | 9.534 | 0.000 | 0.000 | 36.012 |
| AC091516.1 | ENSG00000257752 | 2.022 | -3.040 | 9.523 | 0.000 | 0.000 | 35.914 |
| AC022540.1 | ENSG00000232342 | 2.075 | -5.047 | 9.514 | 0.000 | 0.000 | 35.837 |
| ZBTB40-IT1 | ENSG00000237200 | 2.172 | -1.449 | 9.493 | 0.000 | 0.000 | 35.647 |
| RNU6-553P | ENSG00000200917 | 3.105 | -5.311 | 9.476 | 0.000 | 0.000 | 35.501 |
| RNU1-94P | ENSG00000199497 | 2.785 | -7.241 | 9.465 | 0.000 | 0.000 | 35.400 |
| AC022400.2 | ENSG00000229990 | 2.228 | -5.410 | 9.454 | 0.000 | 0.000 | 35.311 |
| MIR3192 | ENSG00000265137 | 3.071 | -4.256 | 9.447 | 0.000 | 0.000 | 35.245 |
| UBE2V2P3 | ENSG00000238199 | 2.435 | -4.256 | 9.423 | 0.000 | 0.000 | 35.036 |
| AP003108.4 | ENSG00000279632 | 2.082 | -4.451 | 9.410 | 0.000 | 0.000 | 34.923 |
| AC007998.5 | ENSG00000279074 | 2.728 | -5.105 | 9.406 | 0.000 | 0.000 | 34.887 |
| MIR623 | ENSG00000207719 | 2.983 | -6.322 | 9.403 | 0.000 | 0.000 | 34.867 |
| PTTG4P | ENSG00000258571 | 2.565 | -4.500 | 9.392 | 0.000 | 0.000 | 34.772 |
| AC096720.1 | ENSG00000270257 | 2.259 | -0.861 | 9.380 | 0.000 | 0.000 | 34.667 |
| SLC6A1-AS1 | ENSG00000232287 | 2.431 | -5.368 | 9.372 | 0.000 | 0.000 | 34.598 |
| AL590762.4 | ENSG00000229601 | 2.716 | -4.971 | 9.355 | 0.000 | 0.000 | 34.447 |
| RNU6ATAC24P | ENSG00000252620 | 2.591 | -1.696 | 9.354 | 0.000 | 0.000 | 34.439 |
| RAD17P1 | ENSG00000232400 | 2.524 | -1.890 | 9.346 | 0.000 | 0.000 | 34.371 |
| RNA5SP435 | ENSG00000200914 | 3.003 | -5.797 | 9.339 | 0.000 | 0.000 | 34.311 |
| RNU6-1262P | ENSG00000252026 | 2.974 | -3.137 | 9.336 | 0.000 | 0.000 | 34.284 |
| AL592114.3 | ENSG00000231691 | 2.220 | -2.107 | 9.335 | 0.000 | 0.000 | 34.272 |
| AC091053.2 | ENSG00000254900 | 2.424 | -4.825 | 9.325 | 0.000 | 0.000 | 34.187 |
| RNU6-1048P | ENSG00000200924 | 2.145 | 0.092 | 9.322 | 0.000 | 0.000 | 34.161 |
| SNORA67 | ENSG00000252473 | 2.770 | -2.405 | 9.318 | 0.000 | 0.000 | 34.127 |
| AC068446.1 | ENSG00000237161 | 2.083 | -6.020 | 9.317 | 0.000 | 0.000 | 34.122 |
| TBL1XR1-AS1 | ENSG00000231310 | 2.178 | -4.249 | 9.299 | 0.000 | 0.000 | 33.968 |
| TIMM9P2 | ENSG00000232608 | 2.374 | -1.974 | 9.291 | 0.000 | 0.000 | 33.895 |
| AP001271.1 | ENSG00000227726 | 2.186 | -0.099 | 9.284 | 0.000 | 0.000 | 33.834 |
| MIR4526 | ENSG00000263527 | 3.252 | -4.146 | 9.280 | 0.000 | 0.000 | 33.797 |
| SNORD116-2 | ENSG00000207001 | 2.870 | -7.061 | 9.271 | 0.000 | 0.000 | 33.728 |
| AC079336.7 | ENSG00000280245 | 2.137 | -5.591 | 9.270 | 0.000 | 0.000 | 33.713 |
| RN7SL382P | ENSG00000240723 | 2.628 | -6.223 | 9.262 | 0.000 | 0.000 | 33.650 |
| AC099791.2 | ENSG00000271200 | -2.266 | -3.151 | -9.225 | 0.000 | 0.000 | 33.333 |
| HMGN1P15 | ENSG00000250197 | 2.554 | -6.209 | 9.223 | 0.000 | 0.000 | 33.312 |
| Y_RNA | ENSG00000238713 | 2.924 | -6.050 | 9.199 | 0.000 | 0.000 | 33.112 |
| RN7SL559P | ENSG00000240750 | 2.639 | -5.951 | 9.194 | 0.000 | 0.000 | 33.067 |
| Y_RNA | ENSG00000206739 | 2.667 | -1.951 | 9.189 | 0.000 | 0.000 | 33.026 |
| MIR553 | ENSG00000207750 | 3.104 | -3.403 | 9.185 | 0.000 | 0.000 | 32.986 |
| AL133481.1 | ENSG00000235426 | 2.383 | -7.033 | 9.159 | 0.000 | 0.000 | 32.770 |
| HSPE1P26 | ENSG00000220867 | 2.638 | -4.554 | 9.153 | 0.000 | 0.000 | 32.719 |
| AC026150.1 | ENSG00000260693 | 2.249 | -5.078 | 9.143 | 0.000 | 0.000 | 32.632 |
| EIF1AX-AS1 | ENSG00000225037 | 2.473 | -4.504 | 9.143 | 0.000 | 0.000 | 32.628 |
| AL031281.1 | ENSG00000271428 | 2.364 | -4.361 | 9.101 | 0.000 | 0.000 | 32.273 |
| THOC7-AS1 | ENSG00000240549 | 2.266 | -5.454 | 9.086 | 0.000 | 0.000 | 32.148 |
| Y_RNA | ENSG00000252759 | 2.662 | -1.526 | 9.082 | 0.000 | 0.000 | 32.117 |
| AC017101.1 | ENSG00000227227 | 2.370 | -6.165 | 9.070 | 0.000 | 0.000 | 32.018 |
| AC007954.1 | ENSG00000258510 | 2.268 | -7.608 | 9.048 | 0.000 | 0.000 | 31.827 |
| AC110285.6 | ENSG00000275966 | -2.294 | -2.618 | -9.041 | 0.000 | 0.000 | 31.772 |
| AC092574.2 | ENSG00000281016 | 2.055 | -3.773 | 9.041 | 0.000 | 0.000 | 31.768 |
| PRICKLE2-AS2 | ENSG00000241101 | 2.393 | -6.784 | 9.007 | 0.000 | 0.000 | 31.487 |
| RN7SL127P | ENSG00000242175 | 2.474 | -7.330 | 8.999 | 0.000 | 0.000 | 31.415 |
| AC117383.1 | ENSG00000249417 | 2.345 | -3.728 | 8.992 | 0.000 | 0.000 | 31.362 |
| AC018410.1 | ENSG00000256746 | 2.205 | -3.941 | 8.978 | 0.000 | 0.000 | 31.246 |
| AC010261.1 | ENSG00000250882 | 2.443 | -6.369 | 8.976 | 0.000 | 0.000 | 31.229 |
| AC138965.2 | ENSG00000250820 | 2.357 | -7.130 | 8.950 | 0.000 | 0.000 | 31.009 |
| AC105129.3 | ENSG00000270690 | 2.348 | -5.521 | 8.933 | 0.000 | 0.000 | 30.869 |
| RNA5SP310 | ENSG00000239152 | 2.552 | -7.679 | 8.933 | 0.000 | 0.000 | 30.868 |
| AC025048.6 | ENSG00000280852 | 2.058 | -3.468 | 8.923 | 0.000 | 0.000 | 30.787 |
| AC026801.1 | ENSG00000241829 | 2.292 | -3.259 | 8.923 | 0.000 | 0.000 | 30.785 |
| AC134050.1 | ENSG00000241537 | 2.070 | -7.125 | 8.912 | 0.000 | 0.000 | 30.697 |
| Y_RNA | ENSG00000207370 | 2.820 | -3.470 | 8.906 | 0.000 | 0.000 | 30.642 |
| RNU6-26P | ENSG00000206712 | 2.826 | -3.087 | 8.883 | 0.000 | 0.000 | 30.455 |
| AC098848.1 | ENSG00000267112 | 2.139 | -3.201 | 8.864 | 0.000 | 0.000 | 30.296 |
| MIR215 | ENSG00000207590 | 2.738 | -6.730 | 8.863 | 0.000 | 0.000 | 30.285 |
| AP001646.1 | ENSG00000254441 | 2.137 | -5.873 | 8.863 | 0.000 | 0.000 | 30.285 |
| Y_RNA | ENSG00000206651 | 2.842 | -3.360 | 8.849 | 0.000 | 0.000 | 30.175 |
| RNA5SP217 | ENSG00000251768 | 2.909 | -3.461 | 8.844 | 0.000 | 0.000 | 30.128 |
| AC090666.1 | ENSG00000264296 | 2.321 | -4.379 | 8.834 | 0.000 | 0.000 | 30.051 |
| RNU6-828P | ENSG00000201746 | 2.853 | -4.465 | 8.831 | 0.000 | 0.000 | 30.026 |
| Z98048.1 | ENSG00000230769 | 2.300 | -5.194 | 8.813 | 0.000 | 0.000 | 29.875 |
| AC009365.2 | ENSG00000227197 | 2.079 | -7.755 | 8.810 | 0.000 | 0.000 | 29.855 |
| RN7SL246P | ENSG00000265093 | 2.477 | -6.671 | 8.810 | 0.000 | 0.000 | 29.854 |
| AP002373.2 | ENSG00000256533 | 2.056 | -5.516 | 8.800 | 0.000 | 0.000 | 29.772 |
| RNU6-195P | ENSG00000223284 | 2.673 | -2.570 | 8.778 | 0.000 | 0.000 | 29.590 |
| U3 | ENSG00000271817 | 2.222 | -1.876 | 8.773 | 0.000 | 0.000 | 29.546 |
| AC005495.1 | ENSG00000267361 | 2.129 | -5.520 | 8.772 | 0.000 | 0.000 | 29.538 |
| AC116424.1 | ENSG00000250129 | 2.179 | -5.595 | 8.770 | 0.000 | 0.000 | 29.520 |
| RNA5SP465 | ENSG00000212497 | 2.848 | -5.101 | 8.762 | 0.000 | 0.000 | 29.455 |
| AC069257.1 | ENSG00000228028 | 2.272 | -4.106 | 8.743 | 0.000 | 0.000 | 29.304 |
| VN1R80P | ENSG00000268598 | 2.351 | -4.407 | 8.729 | 0.000 | 0.000 | 29.192 |
| RNU1-73P | ENSG00000206698 | 2.628 | -6.415 | 8.718 | 0.000 | 0.000 | 29.096 |
| RNA5SP37 | ENSG00000202290 | 2.402 | -0.775 | 8.716 | 0.000 | 0.000 | 29.084 |
| Y_RNA | ENSG00000201134 | 2.328 | -0.511 | 8.700 | 0.000 | 0.000 | 28.955 |
| AL161757.2 | ENSG00000258428 | 2.244 | -4.510 | 8.698 | 0.000 | 0.000 | 28.940 |
| RN7SL535P | ENSG00000239419 | 2.082 | -1.864 | 8.696 | 0.000 | 0.000 | 28.919 |
| RERG-AS1 | ENSG00000255660 | 2.593 | -4.598 | 8.691 | 0.000 | 0.000 | 28.883 |
| RPL12P44 | ENSG00000227123 | 2.155 | -4.174 | 8.687 | 0.000 | 0.000 | 28.847 |
| AC013714.1 | ENSG00000254606 | 2.191 | -6.446 | 8.669 | 0.000 | 0.000 | 28.698 |
| Y_RNA | ENSG00000200065 | 2.708 | -6.832 | 8.665 | 0.000 | 0.000 | 28.666 |
| MIR5008 | ENSG00000264483 | 2.720 | -6.824 | 8.664 | 0.000 | 0.000 | 28.660 |
| SNORA12 | ENSG00000212175 | 2.063 | -0.710 | 8.660 | 0.000 | 0.000 | 28.629 |
| MIR4740 | ENSG00000266392 | -2.678 | -0.845 | -8.660 | 0.000 | 0.000 | 28.625 |
| RBAKDN | ENSG00000273313 | -2.492 | -5.322 | -8.613 | 0.000 | 0.000 | 28.250 |
| RNA5SP311 | ENSG00000238405 | 2.691 | -2.524 | 8.599 | 0.000 | 0.000 | 28.138 |
| PSMA2P1 | ENSG00000254582 | 2.125 | -7.413 | 8.599 | 0.000 | 0.000 | 28.133 |
| GPR79 | ENSG00000232374 | 2.102 | -6.336 | 8.592 | 0.000 | 0.000 | 28.077 |
| Y_RNA | ENSG00000207195 | 2.827 | -4.004 | 8.588 | 0.000 | 0.000 | 28.048 |
| AC127540.1 | ENSG00000266803 | 2.178 | -6.158 | 8.587 | 0.000 | 0.000 | 28.036 |
| Y_RNA | ENSG00000199740 | 2.426 | -7.893 | 8.582 | 0.000 | 0.000 | 28.000 |
| RNY1P12 | ENSG00000201121 | 2.533 | -7.414 | 8.575 | 0.000 | 0.000 | 27.946 |
| AL132765.1 | ENSG00000230495 | 2.042 | -3.588 | 8.568 | 0.000 | 0.000 | 27.890 |
| AL731892.1 | ENSG00000238145 | 2.348 | -4.740 | 8.567 | 0.000 | 0.000 | 27.876 |
| RN7SL650P | ENSG00000274475 | 2.447 | -4.689 | 8.565 | 0.000 | 0.000 | 27.865 |
| RNU6-540P | ENSG00000207352 | 2.560 | -7.399 | 8.565 | 0.000 | 0.000 | 27.865 |
| AC008443.7 | ENSG00000274525 | 2.226 | -2.329 | 8.554 | 0.000 | 0.000 | 27.771 |
| RNU6-748P | ENSG00000207378 | 2.753 | -5.743 | 8.519 | 0.000 | 0.000 | 27.497 |
| AP000753.2 | ENSG00000256789 | 2.272 | -4.665 | 8.504 | 0.000 | 0.000 | 27.378 |
| AC113146.1 | ENSG00000259423 | 2.050 | -5.365 | 8.503 | 0.000 | 0.000 | 27.371 |
| AC018620.1 | ENSG00000254273 | 2.345 | -6.367 | 8.502 | 0.000 | 0.000 | 27.357 |
| MIR4782 | ENSG00000265429 | 2.698 | -4.128 | 8.465 | 0.000 | 0.000 | 27.064 |
| AL031667.2 | ENSG00000235088 | 2.408 | -5.377 | 8.464 | 0.000 | 0.000 | 27.060 |
| AP000654.1 | ENSG00000269895 | 2.037 | -5.835 | 8.463 | 0.000 | 0.000 | 27.051 |
| RNU7-75P | ENSG00000251880 | 2.915 | -2.565 | 8.453 | 0.000 | 0.000 | 26.973 |
| RNU6-720P | ENSG00000252172 | 2.747 | -4.388 | 8.434 | 0.000 | 0.000 | 26.824 |
| FAM60BP | ENSG00000263829 | 2.053 | -4.644 | 8.430 | 0.000 | 0.000 | 26.786 |
| AC007386.1 | ENSG00000227394 | 2.081 | -4.297 | 8.426 | 0.000 | 0.000 | 26.759 |
| DUTP7 | ENSG00000250473 | 2.110 | -7.637 | 8.423 | 0.000 | 0.000 | 26.733 |
| AC104564.2 | ENSG00000263370 | 2.401 | -4.405 | 8.420 | 0.000 | 0.000 | 26.706 |
| AL136298.3 | ENSG00000259086 | 2.087 | -4.487 | 8.416 | 0.000 | 0.000 | 26.676 |
| RNY1P13 | ENSG00000201900 | 2.448 | -1.791 | 8.410 | 0.000 | 0.000 | 26.634 |
| SNORD116-1 | ENSG00000207063 | 2.457 | -7.795 | 8.406 | 0.000 | 0.000 | 26.602 |
| RNU6-466P | ENSG00000212526 | 2.385 | -7.905 | 8.397 | 0.000 | 0.000 | 26.530 |
| RNU6-196P | ENSG00000207042 | 2.662 | -6.120 | 8.396 | 0.000 | 0.000 | 26.519 |
| RNA5SP290 | ENSG00000252942 | 2.698 | -5.388 | 8.385 | 0.000 | 0.000 | 26.432 |
| AC104187.1 | ENSG00000271937 | -2.042 | -4.337 | -8.374 | 0.000 | 0.000 | 26.346 |
| AC087752.2 | ENSG00000253528 | 2.170 | -2.429 | 8.366 | 0.000 | 0.000 | 26.282 |
| AL021997.2 | ENSG00000273712 | 2.061 | -1.852 | 8.360 | 0.000 | 0.000 | 26.238 |
| AL139081.1 | ENSG00000232986 | 2.456 | -5.011 | 8.359 | 0.000 | 0.000 | 26.225 |
| RNU6-577P | ENSG00000252756 | 2.712 | -6.166 | 8.358 | 0.000 | 0.000 | 26.221 |
| AC068790.6 | ENSG00000270095 | 2.314 | -3.578 | 8.354 | 0.000 | 0.000 | 26.192 |
| AC231657.2 | ENSG00000280116 | 2.455 | -4.795 | 8.346 | 0.000 | 0.000 | 26.127 |
| RN7SL862P | ENSG00000242651 | 2.377 | -6.830 | 8.341 | 0.000 | 0.000 | 26.088 |
| Y_RNA | ENSG00000199331 | 2.239 | -0.897 | 8.325 | 0.000 | 0.000 | 25.960 |
| SNORA47 | ENSG00000238961 | 2.590 | -3.467 | 8.324 | 0.000 | 0.000 | 25.957 |
| AC073167.1 | ENSG00000259589 | 2.087 | -7.040 | 8.317 | 0.000 | 0.000 | 25.903 |
| SRP68P1 | ENSG00000266129 | 2.487 | -6.710 | 8.312 | 0.000 | 0.000 | 25.863 |
| LYST-AS1 | ENSG00000229463 | 2.226 | -5.826 | 8.309 | 0.000 | 0.000 | 25.835 |
| DUTP2 | ENSG00000254388 | 2.201 | -4.099 | 8.299 | 0.000 | 0.000 | 25.759 |
| Y_RNA | ENSG00000202523 | 2.719 | -5.799 | 8.295 | 0.000 | 0.000 | 25.732 |
| RNU6-1165P | ENSG00000222051 | 2.626 | -3.084 | 8.280 | 0.000 | 0.000 | 25.616 |
| Y_RNA | ENSG00000207342 | 2.326 | -0.451 | 8.276 | 0.000 | 0.000 | 25.580 |
| AC022167.4 | ENSG00000261481 | -2.106 | -4.485 | -8.275 | 0.000 | 0.000 | 25.571 |
| AC022973.1 | ENSG00000243402 | 2.161 | -6.190 | 8.260 | 0.000 | 0.000 | 25.459 |
| MIR125B1 | ENSG00000207971 | 2.444 | -7.715 | 8.235 | 0.000 | 0.000 | 25.262 |
| MIR550A3 | ENSG00000212024 | 2.653 | -6.377 | 8.231 | 0.000 | 0.000 | 25.229 |
| AC018511.2 | ENSG00000234149 | 2.179 | -8.160 | 8.224 | 0.000 | 0.000 | 25.176 |
| U82695.1 | ENSG00000224963 | -2.134 | -5.756 | -8.220 | 0.000 | 0.000 | 25.145 |
| RNU6-1005P | ENSG00000207248 | 2.674 | -4.872 | 8.218 | 0.000 | 0.000 | 25.135 |
| AC104806.1 | ENSG00000250030 | 2.303 | -5.242 | 8.215 | 0.000 | 0.000 | 25.105 |
| LLPHP2 | ENSG00000235514 | 2.091 | -7.686 | 8.210 | 0.000 | 0.000 | 25.070 |
| SNRPGP9 | ENSG00000228551 | -2.215 | -2.570 | -8.209 | 0.000 | 0.000 | 25.064 |
| ARMC2-AS1 | ENSG00000230290 | 2.708 | -4.897 | 8.206 | 0.000 | 0.000 | 25.038 |
| AC022296.3 | ENSG00000272832 | -2.278 | -5.582 | -8.199 | 0.000 | 0.000 | 24.987 |
| SNORA80E | ENSG00000207475 | 2.439 | -2.553 | 8.180 | 0.000 | 0.000 | 24.840 |
| AC027544.1 | ENSG00000256361 | 2.086 | -4.055 | 8.169 | 0.000 | 0.000 | 24.753 |
| RNU6-915P | ENSG00000201813 | 2.500 | -7.133 | 8.168 | 0.000 | 0.000 | 24.749 |
| PFN1P8 | ENSG00000244371 | 2.144 | -5.451 | 8.159 | 0.000 | 0.000 | 24.676 |
| RNU7-49P | ENSG00000251991 | 2.187 | 0.375 | 8.157 | 0.000 | 0.000 | 24.663 |
| AC008937.2 | ENSG00000237705 | 2.087 | -3.913 | 8.155 | 0.000 | 0.000 | 24.648 |
| DDTP1 | ENSG00000230683 | 2.223 | -6.591 | 8.153 | 0.000 | 0.000 | 24.629 |
| Y_RNA | ENSG00000201563 | 2.699 | -4.872 | 8.152 | 0.000 | 0.000 | 24.623 |
| Y_RNA | ENSG00000201988 | 2.198 | -0.768 | 8.141 | 0.000 | 0.000 | 24.541 |
| AC013652.2 | ENSG00000259447 | 2.149 | -6.664 | 8.136 | 0.000 | 0.000 | 24.499 |
| RNU6-1157P | ENSG00000207185 | 2.504 | -2.707 | 8.134 | 0.000 | 0.000 | 24.482 |
| RNU2-69P | ENSG00000251870 | 2.553 | -6.781 | 8.132 | 0.000 | 0.000 | 24.466 |
| AL928711.1 | ENSG00000259984 | 2.093 | -4.232 | 8.128 | 0.000 | 0.000 | 24.442 |
| SBSN | ENSG00000189001 | -2.060 | -2.497 | -8.128 | 0.000 | 0.000 | 24.437 |
| AC026407.1 | ENSG00000253630 | 2.228 | -7.539 | 8.127 | 0.000 | 0.000 | 24.428 |
| AC096708.3 | ENSG00000264472 | 2.072 | -6.994 | 8.121 | 0.000 | 0.000 | 24.385 |
| RNU6-1024P | ENSG00000206926 | 2.563 | -3.308 | 8.112 | 0.000 | 0.000 | 24.319 |
| AC007347.1 | ENSG00000261049 | 2.247 | -3.769 | 8.108 | 0.000 | 0.000 | 24.283 |
| AC025423.1 | ENSG00000256325 | 2.224 | -4.703 | 8.099 | 0.000 | 0.000 | 24.215 |
| MIR30C2 | ENSG00000199094 | 2.234 | 0.813 | 8.085 | 0.000 | 0.000 | 24.112 |
| NCKAP5-IT1 | ENSG00000232474 | 2.128 | -6.764 | 8.084 | 0.000 | 0.000 | 24.103 |
| AF213884.1 | ENSG00000241981 | 2.088 | -7.277 | 8.076 | 0.000 | 0.000 | 24.038 |
| AC092471.1 | ENSG00000255669 | 2.060 | -5.100 | 8.051 | 0.000 | 0.000 | 23.855 |
| AL022238.1 | ENSG00000227413 | 2.244 | -4.545 | 8.049 | 0.000 | 0.000 | 23.836 |
| AL356776.1 | ENSG00000217512 | 2.116 | -7.102 | 8.035 | 0.000 | 0.000 | 23.733 |
| MIR579 | ENSG00000207956 | 2.648 | -4.452 | 8.028 | 0.000 | 0.000 | 23.676 |
| OR4K12P | ENSG00000218549 | 2.746 | -4.877 | 8.027 | 0.000 | 0.000 | 23.667 |
| MIR548V | ENSG00000265520 | 2.535 | -7.249 | 8.026 | 0.000 | 0.000 | 23.662 |
| AC004024.1 | ENSG00000257624 | 2.005 | -2.093 | 8.024 | 0.000 | 0.000 | 23.650 |
| RPL21P32 | ENSG00000224019 | 2.181 | -5.267 | 8.023 | 0.000 | 0.000 | 23.639 |
| AC138305.3 | ENSG00000279225 | -2.236 | -3.238 | -8.022 | 0.000 | 0.000 | 23.633 |
| AC090515.5 | ENSG00000259402 | 2.093 | -5.461 | 8.021 | 0.000 | 0.000 | 23.621 |
| AC005828.1 | ENSG00000226797 | 2.150 | -5.411 | 8.017 | 0.000 | 0.000 | 23.598 |
| L29074.1 | ENSG00000227083 | 2.094 | -4.474 | 8.002 | 0.000 | 0.000 | 23.478 |
| AC027698.1 | ENSG00000253116 | 2.098 | -4.337 | 7.986 | 0.000 | 0.000 | 23.358 |
| Y_RNA | ENSG00000274967 | 2.577 | -3.997 | 7.983 | 0.000 | 0.000 | 23.334 |
| UBE2CP1 | ENSG00000258648 | 2.092 | -5.084 | 7.961 | 0.000 | 0.000 | 23.174 |
| AL358913.2 | ENSG00000258894 | 2.155 | -6.629 | 7.957 | 0.000 | 0.000 | 23.145 |
| AC010401.1 | ENSG00000242307 | 2.216 | -5.977 | 7.923 | 0.000 | 0.000 | 22.887 |
| AC020910.1 | ENSG00000268683 | 2.194 | -5.654 | 7.912 | 0.000 | 0.000 | 22.804 |
| AC099778.2 | ENSG00000276925 | 2.111 | -2.294 | 7.911 | 0.000 | 0.000 | 22.801 |
| ATP5EP1 | ENSG00000250922 | 2.261 | -7.452 | 7.903 | 0.000 | 0.000 | 22.736 |
| AL096712.1 | ENSG00000275550 | 2.461 | -5.710 | 7.896 | 0.000 | 0.000 | 22.688 |
| RNU4-82P | ENSG00000199313 | 2.463 | -3.953 | 7.877 | 0.000 | 0.000 | 22.541 |
| RN7SKP275 | ENSG00000201496 | 2.465 | -4.026 | 7.875 | 0.000 | 0.000 | 22.532 |
| RPL30P2 | ENSG00000234272 | 2.242 | -4.873 | 7.864 | 0.000 | 0.000 | 22.448 |
| RN7SKP271 | ENSG00000222460 | 2.006 | -2.954 | 7.862 | 0.000 | 0.000 | 22.431 |
| MIR4482 | ENSG00000266852 | 2.071 | 0.017 | 7.861 | 0.000 | 0.000 | 22.424 |
| RPS23P6 | ENSG00000241641 | -2.006 | -3.347 | -7.848 | 0.000 | 0.000 | 22.331 |
| AC008417.1 | ENSG00000249068 | 2.027 | -5.628 | 7.841 | 0.000 | 0.000 | 22.276 |
| AC091544.4 | ENSG00000259064 | 2.163 | -4.161 | 7.838 | 0.000 | 0.000 | 22.254 |
| ACA59 | ENSG00000251775 | 2.029 | -1.189 | 7.838 | 0.000 | 0.000 | 22.254 |
| RN7SKP219 | ENSG00000252634 | 2.028 | -8.175 | 7.836 | 0.000 | 0.000 | 22.242 |
| AC126615.2 | ENSG00000257121 | 2.078 | -6.299 | 7.836 | 0.000 | 0.000 | 22.241 |
| RNA5SP491 | ENSG00000199806 | 2.369 | -7.191 | 7.836 | 0.000 | 0.000 | 22.237 |
| AC010378.1 | ENSG00000275506 | 2.013 | -3.500 | 7.822 | 0.000 | 0.000 | 22.132 |
| MIR193A | ENSG00000207614 | -2.646 | -3.687 | -7.816 | 0.000 | 0.000 | 22.094 |
| STH | ENSG00000256762 | 2.265 | -4.182 | 7.813 | 0.000 | 0.000 | 22.068 |
| AC124312.6 | ENSG00000279735 | 2.026 | -7.510 | 7.809 | 0.000 | 0.000 | 22.039 |
| MIR4731 | ENSG00000265110 | 2.356 | -7.862 | 7.807 | 0.000 | 0.000 | 22.028 |
| RNU6-795P | ENSG00000252132 | 2.478 | -6.533 | 7.806 | 0.000 | 0.000 | 22.020 |
| Metazoa_SRP | ENSG00000277371 | 2.065 | -3.361 | 7.804 | 0.000 | 0.000 | 22.004 |
| RNA5SP268 | ENSG00000252637 | 2.555 | -4.720 | 7.795 | 0.000 | 0.000 | 21.938 |
| RNU2-28P | ENSG00000222389 | 2.197 | -7.398 | 7.776 | 0.000 | 0.000 | 21.797 |
| AC025576.2 | ENSG00000255817 | 2.188 | -6.052 | 7.758 | 0.000 | 0.000 | 21.664 |
| MIR3149 | ENSG00000266712 | 2.324 | -2.228 | 7.757 | 0.000 | 0.000 | 21.659 |
| CBX5P1 | ENSG00000241535 | 2.044 | -5.639 | 7.745 | 0.000 | 0.000 | 21.572 |
| CHCHD3P1 | ENSG00000236420 | 2.265 | -5.597 | 7.738 | 0.000 | 0.000 | 21.520 |
| RNA5SP385 | ENSG00000251756 | 2.225 | -7.791 | 7.712 | 0.000 | 0.000 | 21.329 |
| FTH1P24 | ENSG00000249302 | 2.057 | -6.899 | 7.711 | 0.000 | 0.000 | 21.322 |
| AC010275.1 | ENSG00000248268 | 2.096 | -5.803 | 7.674 | 0.000 | 0.000 | 21.054 |
| RHOT1P2 | ENSG00000203616 | 2.343 | -4.142 | 7.673 | 0.000 | 0.000 | 21.046 |
| RANP3 | ENSG00000254500 | 2.070 | -7.368 | 7.673 | 0.000 | 0.000 | 21.046 |
| AC009303.1 | ENSG00000224967 | 2.120 | -5.463 | 7.667 | 0.000 | 0.000 | 21.002 |
| AL022345.3 | ENSG00000277479 | 2.252 | -4.059 | 7.667 | 0.000 | 0.000 | 21.001 |
| AC018511.1 | ENSG00000227186 | 2.150 | -5.617 | 7.667 | 0.000 | 0.000 | 20.999 |
| RN7SL314P | ENSG00000244402 | 2.940 | -0.345 | 7.664 | 0.000 | 0.000 | 20.976 |
| SPIN4-AS1 | ENSG00000233661 | 2.119 | -5.888 | 7.658 | 0.000 | 0.000 | 20.932 |
| MYCBP2-AS2 | ENSG00000229521 | 2.096 | -5.639 | 7.656 | 0.000 | 0.000 | 20.923 |
| AL591848.1 | ENSG00000228879 | 2.228 | -4.963 | 7.645 | 0.000 | 0.000 | 20.840 |
| AL355102.1 | ENSG00000258412 | 2.109 | -5.856 | 7.641 | 0.000 | 0.000 | 20.809 |
| MIR3165 | ENSG00000263742 | 2.716 | -5.281 | 7.639 | 0.000 | 0.000 | 20.798 |
| PFN1P4 | ENSG00000225080 | 2.062 | -4.634 | 7.638 | 0.000 | 0.000 | 20.792 |
| RNU6-402P | ENSG00000222610 | 2.102 | -8.286 | 7.627 | 0.000 | 0.000 | 20.710 |
| AL354726.1 | ENSG00000236896 | 2.019 | -4.112 | 7.620 | 0.000 | 0.000 | 20.657 |
| MYB-AS1 | ENSG00000236703 | 2.037 | -2.678 | 7.609 | 0.000 | 0.000 | 20.577 |
| SNORA72 | ENSG00000201944 | 2.251 | -2.302 | 7.603 | 0.000 | 0.000 | 20.536 |
| AC093297.1 | ENSG00000248779 | 2.934 | -1.622 | 7.602 | 0.000 | 0.000 | 20.529 |
| Metazoa_SRP | ENSG00000276735 | 2.042 | -3.869 | 7.592 | 0.000 | 0.000 | 20.457 |
| SNORA62 | ENSG00000202374 | 2.335 | -6.394 | 7.581 | 0.000 | 0.000 | 20.380 |
| Metazoa_SRP | ENSG00000276359 | 2.034 | -7.959 | 7.580 | 0.000 | 0.000 | 20.374 |
| Z98752.2 | ENSG00000234271 | 2.173 | -5.950 | 7.573 | 0.000 | 0.000 | 20.319 |
| AC010997.5 | ENSG00000273248 | -2.211 | -6.153 | -7.567 | 0.000 | 0.000 | 20.278 |
| AC126177.5 | ENSG00000257426 | 2.215 | -7.241 | 7.552 | 0.000 | 0.000 | 20.171 |
| FGF7P1 | ENSG00000264061 | 2.550 | -5.320 | 7.547 | 0.000 | 0.000 | 20.136 |
| AC108472.1 | ENSG00000235902 | 2.038 | -3.482 | 7.535 | 0.000 | 0.000 | 20.051 |
| AP000907.3 | ENSG00000255334 | 2.042 | -3.352 | 7.519 | 0.000 | 0.000 | 19.933 |
| Y_RNA | ENSG00000200120 | 2.399 | -6.359 | 7.512 | 0.000 | 0.000 | 19.885 |
| RN7SL762P | ENSG00000244308 | 2.158 | -6.469 | 7.512 | 0.000 | 0.000 | 19.881 |
| RNU6-851P | ENSG00000207295 | 2.277 | -7.085 | 7.508 | 0.000 | 0.000 | 19.853 |
| AL163153.1 | ENSG00000259047 | 2.275 | -7.050 | 7.504 | 0.000 | 0.000 | 19.829 |
| Y_RNA | ENSG00000200591 | 2.491 | -4.463 | 7.502 | 0.000 | 0.000 | 19.811 |
| MIR641 | ENSG00000207631 | 2.445 | -3.283 | 7.496 | 0.000 | 0.000 | 19.767 |
| Y_RNA | ENSG00000200351 | 2.125 | -7.977 | 7.479 | 0.000 | 0.000 | 19.652 |
| RNU1-115P | ENSG00000202199 | 2.209 | -7.471 | 7.477 | 0.000 | 0.000 | 19.633 |
| AC073912.2 | ENSG00000256299 | 2.181 | -4.904 | 7.472 | 0.000 | 0.000 | 19.597 |
| RNU6-335P | ENSG00000201433 | 2.049 | -8.156 | 7.472 | 0.000 | 0.000 | 19.596 |
| AC093838.1 | ENSG00000273588 | 2.618 | -1.641 | 7.455 | 0.000 | 0.000 | 19.475 |
| RNU6-890P | ENSG00000206848 | 2.443 | -4.542 | 7.428 | 0.000 | 0.000 | 19.285 |
| RNA5SP195 | ENSG00000199545 | 2.420 | -5.371 | 7.421 | 0.000 | 0.000 | 19.237 |
| Y_RNA | ENSG00000201071 | 2.188 | -7.586 | 7.412 | 0.000 | 0.000 | 19.173 |
| RNA5SP494 | ENSG00000222585 | 2.370 | -4.168 | 7.399 | 0.000 | 0.000 | 19.081 |
| RNU4-23P | ENSG00000199709 | 2.277 | -2.570 | 7.380 | 0.000 | 0.000 | 18.948 |
| SNORA23 | ENSG00000201998 | 2.166 | -2.539 | 7.367 | 0.000 | 0.000 | 18.857 |
| AC091812.3 | ENSG00000275875 | -2.175 | -6.211 | -7.362 | 0.000 | 0.000 | 18.820 |
| RNY1P4 | ENSG00000207325 | 2.405 | -5.077 | 7.358 | 0.000 | 0.000 | 18.798 |
| AL356157.2 | ENSG00000270767 | 2.271 | -2.596 | 7.355 | 0.000 | 0.000 | 18.775 |
| MIR5192 | ENSG00000266097 | 2.033 | -8.292 | 7.353 | 0.000 | 0.000 | 18.762 |
| SCARNA15 | ENSG00000252218 | 2.365 | -5.044 | 7.333 | 0.000 | 0.000 | 18.618 |
| AL049648.1 | ENSG00000225417 | 2.208 | -5.354 | 7.321 | 0.000 | 0.000 | 18.534 |
| MIR378H | ENSG00000263361 | 2.396 | -2.336 | 7.319 | 0.000 | 0.000 | 18.526 |
| RNA5SP21 | ENSG00000222849 | 2.427 | -5.828 | 7.315 | 0.000 | 0.000 | 18.497 |
| RNU6-32P | ENSG00000206675 | 2.224 | -7.078 | 7.297 | 0.000 | 0.000 | 18.372 |
| RN7SL258P | ENSG00000240589 | 2.084 | -6.234 | 7.297 | 0.000 | 0.000 | 18.371 |
| PSME2P6 | ENSG00000227462 | -2.058 | -4.950 | -7.278 | 0.000 | 0.000 | 18.236 |
| AL021997.1 | ENSG00000270326 | 2.038 | -2.468 | 7.277 | 0.000 | 0.000 | 18.233 |
| RNU6-920P | ENSG00000222858 | 2.104 | -7.895 | 7.269 | 0.000 | 0.000 | 18.178 |
| RNU6-767P | ENSG00000206859 | 2.338 | -6.149 | 7.267 | 0.000 | 0.000 | 18.162 |
| RNU6-288P | ENSG00000200560 | 2.301 | -6.707 | 7.265 | 0.000 | 0.000 | 18.146 |
| AC104058.1 | ENSG00000226410 | 2.013 | -7.250 | 7.243 | 0.000 | 0.000 | 17.995 |
| ZBTB8OSP2 | ENSG00000172799 | -2.080 | -4.329 | -7.208 | 0.000 | 0.000 | 17.755 |
| U4 | ENSG00000278374 | 2.102 | -1.948 | 7.204 | 0.000 | 0.000 | 17.731 |
| SNORA75 | ENSG00000212533 | 2.280 | -4.697 | 7.191 | 0.000 | 0.000 | 17.635 |
| CR392039.4 | ENSG00000279720 | 2.011 | -3.022 | 7.189 | 0.000 | 0.000 | 17.628 |
| MIR491 | ENSG00000207609 | 2.318 | -6.190 | 7.173 | 0.000 | 0.000 | 17.512 |
| SNORA73 | ENSG00000199977 | 2.143 | -6.407 | 7.154 | 0.000 | 0.000 | 17.382 |
| MIR3173 | ENSG00000264607 | 2.044 | -8.203 | 7.125 | 0.000 | 0.000 | 17.190 |
| SNORD116-14 | ENSG00000206621 | 2.055 | -7.995 | 7.122 | 0.000 | 0.000 | 17.168 |
| MIR3146 | ENSG00000265932 | 2.133 | -7.631 | 7.101 | 0.000 | 0.000 | 17.022 |
| RNU6-969P | ENSG00000206627 | 2.234 | -6.523 | 7.100 | 0.000 | 0.000 | 17.018 |
| MIR210 | ENSG00000199038 | -2.390 | -5.420 | -7.095 | 0.000 | 0.000 | 16.981 |
| RNU6-377P | ENSG00000251774 | 2.362 | -4.917 | 7.093 | 0.000 | 0.000 | 16.967 |
| Y_RNA | ENSG00000207231 | 2.301 | -5.402 | 7.079 | 0.000 | 0.000 | 16.878 |
| MRPL57P3 | ENSG00000275940 | 2.101 | -6.672 | 7.078 | 0.000 | 0.000 | 16.866 |
| AC113410.3 | ENSG00000279691 | -2.042 | -3.425 | -7.073 | 0.000 | 0.000 | 16.835 |
| RNA5SP425 | ENSG00000222268 | -2.188 | -7.088 | -7.069 | 0.000 | 0.000 | 16.806 |
| MTCO2P29 | ENSG00000243257 | 2.142 | -6.660 | 7.067 | 0.000 | 0.000 | 16.793 |
| MIR3174 | ENSG00000265871 | 2.404 | -4.936 | 7.057 | 0.000 | 0.000 | 16.726 |
| RNU7-141P | ENSG00000238777 | 2.416 | -6.300 | 7.051 | 0.000 | 0.000 | 16.689 |
| RNA5SP61 | ENSG00000252262 | 2.317 | -6.476 | 7.029 | 0.000 | 0.000 | 16.541 |
| SNORA79B | ENSG00000222489 | 2.156 | -2.986 | 7.022 | 0.000 | 0.000 | 16.490 |
| UNC93B2 | ENSG00000155070 | -2.004 | -4.900 | -6.997 | 0.000 | 0.000 | 16.325 |
| AL355309.1 | ENSG00000214748 | -2.141 | -1.882 | -6.977 | 0.000 | 0.000 | 16.187 |
| C11orf86 | ENSG00000173237 | -2.061 | -6.480 | -6.963 | 0.000 | 0.000 | 16.099 |
| AC044784.2 | ENSG00000234752 | 2.114 | -6.038 | 6.952 | 0.000 | 0.000 | 16.021 |
| MIR4766 | ENSG00000266594 | 2.273 | -6.481 | 6.951 | 0.000 | 0.000 | 16.020 |
| AC093702.1 | ENSG00000231156 | -2.026 | -3.737 | -6.947 | 0.000 | 0.000 | 15.992 |
| RNY4P19 | ENSG00000199400 | 2.294 | -3.246 | 6.920 | 0.000 | 0.000 | 15.812 |
| RNU6-1121P | ENSG00000252611 | 2.204 | -6.115 | 6.908 | 0.000 | 0.000 | 15.730 |
| Y_RNA | ENSG00000252621 | 2.944 | 1.583 | 6.903 | 0.000 | 0.000 | 15.698 |
| SNORD117 | ENSG00000201785 | 2.110 | -1.247 | 6.888 | 0.000 | 0.000 | 15.599 |
| RNU1-124P | ENSG00000200731 | 2.152 | -6.296 | 6.888 | 0.000 | 0.000 | 15.599 |
| Y_RNA | ENSG00000207467 | 2.076 | -7.305 | 6.873 | 0.000 | 0.000 | 15.500 |
| Y_RNA | ENSG00000199875 | 2.192 | -6.222 | 6.865 | 0.000 | 0.000 | 15.451 |
| Y_RNA | ENSG00000199471 | 2.172 | -2.656 | 6.848 | 0.000 | 0.000 | 15.336 |
| RNU6-807P | ENSG00000252614 | 2.153 | -3.217 | 6.838 | 0.000 | 0.000 | 15.275 |
| SEPT14P8 | ENSG00000255464 | 2.021 | -6.765 | 6.832 | 0.000 | 0.000 | 15.231 |
| RNU4-89P | ENSG00000272359 | 2.126 | -3.378 | 6.827 | 0.000 | 0.000 | 15.202 |
| MIR3127 | ENSG00000264157 | 2.103 | -6.971 | 6.827 | 0.000 | 0.000 | 15.202 |
| Y_RNA | ENSG00000223188 | 2.260 | -4.518 | 6.813 | 0.000 | 0.000 | 15.111 |
| AL161931.1 | ENSG00000275675 | 2.014 | -5.783 | 6.781 | 0.000 | 0.000 | 14.898 |
| RNU7-3P | ENSG00000252244 | 2.317 | -2.990 | 6.780 | 0.000 | 0.000 | 14.891 |
| MIR5587 | ENSG00000266124 | 2.189 | -0.823 | 6.765 | 0.000 | 0.000 | 14.796 |
| TRAJ10 | ENSG00000211879 | 2.168 | -7.209 | 6.723 | 0.000 | 0.000 | 14.528 |
| SNORD62A | ENSG00000235284 | 2.206 | -6.226 | 6.711 | 0.000 | 0.000 | 14.446 |
| MIR4744 | ENSG00000263849 | 2.160 | -6.657 | 6.671 | 0.000 | 0.000 | 14.192 |
| RNA5SP434 | ENSG00000252456 | 2.008 | -7.213 | 6.666 | 0.000 | 0.000 | 14.158 |
| RNU6-1285P | ENSG00000200350 | 2.089 | -6.837 | 6.664 | 0.000 | 0.000 | 14.149 |
| RNA5SP299 | ENSG00000212505 | 2.389 | -4.919 | 6.644 | 0.000 | 0.000 | 14.021 |
| RNU6-875P | ENSG00000252297 | 2.230 | -4.493 | 6.596 | 0.000 | 0.000 | 13.714 |
| AL022718.1 | ENSG00000226532 | -2.250 | -3.985 | -6.589 | 0.000 | 0.000 | 13.669 |
| Y_RNA | ENSG00000206582 | 2.201 | -5.068 | 6.584 | 0.000 | 0.000 | 13.637 |
| AC092755.1 | ENSG00000227161 | 2.158 | -5.728 | 6.576 | 0.000 | 0.000 | 13.587 |
| SNORD67 | ENSG00000212135 | 2.143 | -3.493 | 6.549 | 0.000 | 0.000 | 13.417 |
| HLA-Z | ENSG00000235301 | 2.163 | -6.357 | 6.549 | 0.000 | 0.000 | 13.415 |
| MIR8055 | ENSG00000276580 | 2.162 | -4.704 | 6.541 | 0.000 | 0.000 | 13.364 |
| SNORA22B | ENSG00000206603 | 2.039 | -3.669 | 6.536 | 0.000 | 0.000 | 13.334 |
| AC069503.3 | ENSG00000278084 | 2.008 | -6.339 | 6.532 | 0.000 | 0.000 | 13.307 |
| MIR544B | ENSG00000265981 | 2.112 | -6.747 | 6.528 | 0.000 | 0.000 | 13.282 |
| RNU6-863P | ENSG00000251798 | 2.176 | -5.254 | 6.491 | 0.000 | 0.000 | 13.055 |
| Y_RNA | ENSG00000202279 | 2.162 | -5.289 | 6.481 | 0.000 | 0.000 | 12.989 |
| FTX_1 | ENSG00000278421 | 2.260 | -4.352 | 6.472 | 0.000 | 0.000 | 12.936 |
| Y_RNA | ENSG00000252874 | 2.012 | -2.269 | 6.471 | 0.000 | 0.000 | 12.925 |
| RNU6-1079P | ENSG00000199731 | 2.112 | -5.042 | 6.451 | 0.000 | 0.000 | 12.804 |
| ANKRD30A | ENSG00000148513 | 2.051 | 1.939 | 6.435 | 0.000 | 0.000 | 12.707 |
| Y_RNA | ENSG00000199890 | 2.075 | -5.802 | 6.368 | 0.000 | 0.000 | 12.294 |
| RNA5SP345 | ENSG00000222578 | 2.004 | -6.535 | 6.368 | 0.000 | 0.000 | 12.289 |
| RNA5SP132 | ENSG00000201595 | 2.063 | -4.915 | 6.345 | 0.000 | 0.000 | 12.154 |
| MIR10B | ENSG00000207744 | 2.112 | -3.441 | 6.315 | 0.000 | 0.000 | 11.967 |
| AL132708.1 | ENSG00000256357 | 2.038 | -3.882 | 6.311 | 0.000 | 0.000 | 11.944 |
| snoU2_19 | ENSG00000201592 | 2.024 | -7.064 | 6.300 | 0.000 | 0.000 | 11.876 |
| MIR148B | ENSG00000199122 | 2.017 | -5.997 | 6.226 | 0.000 | 0.000 | 11.432 |
| MIR563 | ENSG00000207815 | 2.035 | -6.717 | 6.225 | 0.000 | 0.000 | 11.421 |
| MIR3136 | ENSG00000265355 | 2.008 | -6.703 | 6.210 | 0.000 | 0.000 | 11.334 |
| MIR556 | ENSG00000207729 | 2.048 | -5.883 | 6.109 | 0.000 | 0.000 | 10.735 |
| VN1R53P | ENSG00000236313 | 2.150 | -0.299 | 6.028 | 0.000 | 0.000 | 10.262 |
| AL358913.1 | ENSG00000258387 | 2.321 | -3.485 | 5.994 | 0.000 | 0.000 | 10.064 |
| SNORD53B | ENSG00000265706 | 2.045 | -4.554 | 5.920 | 0.000 | 0.000 | 9.638 |
| AC044784.1 | ENSG00000223808 | 2.089 | 0.885 | 5.913 | 0.000 | 0.000 | 9.597 |
| MIR6859-1 | ENSG00000278267 | -2.039 | -3.826 | -5.851 | 0.000 | 0.000 | 9.244 |
| LINC02224 | ENSG00000249203 | 2.114 | -1.899 | 5.627 | 0.000 | 0.000 | 7.994 |
| PSPHP1 | ENSG00000226278 | -2.226 | -1.825 | -4.411 | 0.000 | 0.000 | 2.040 |
