# Supplementary figures and images for: A Histone Acetylation Modulator Gene Signature for Classification and Prognosis of Breast Cancer
Source: Curr Oncol. 2021 Feb 17;28(1):928–39. doi: 10.3390/curroncol28010091 (PMC7985767; doi:10.3390/curroncol28010091)

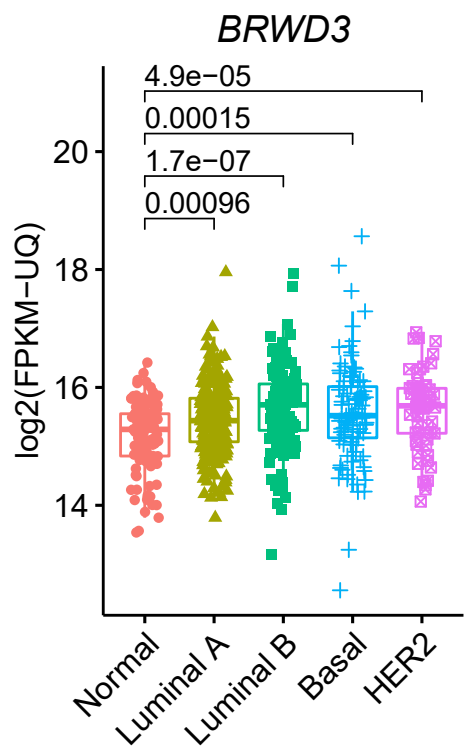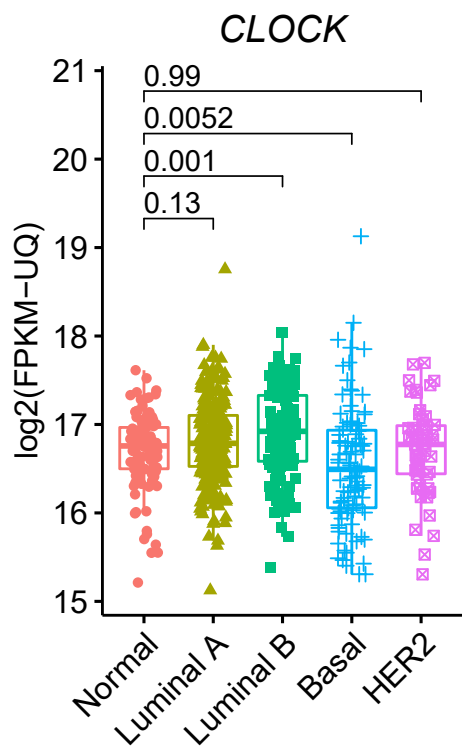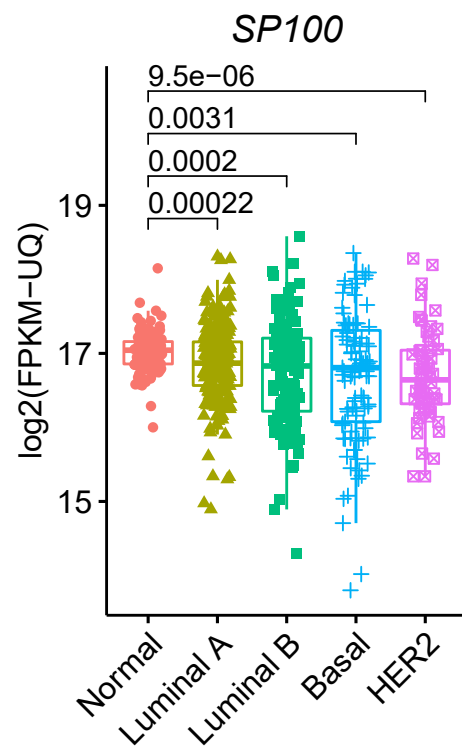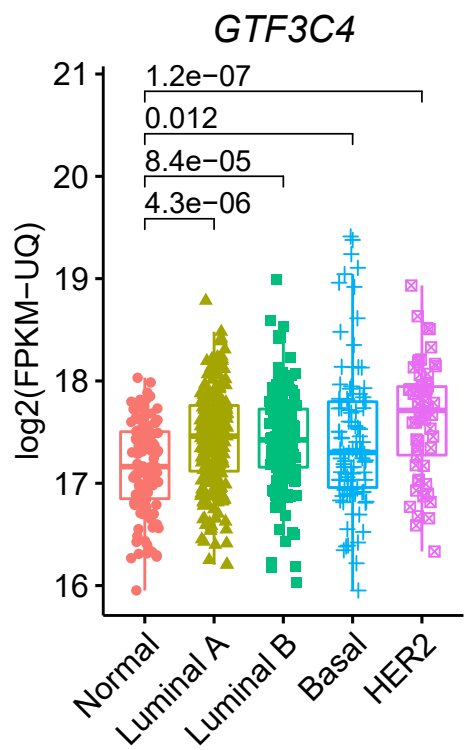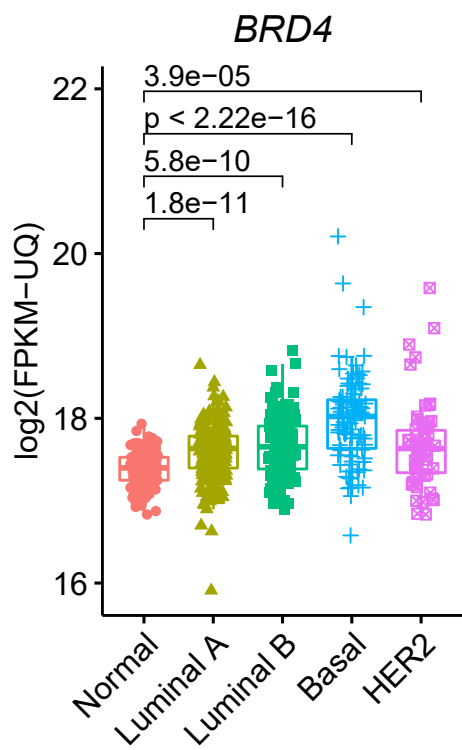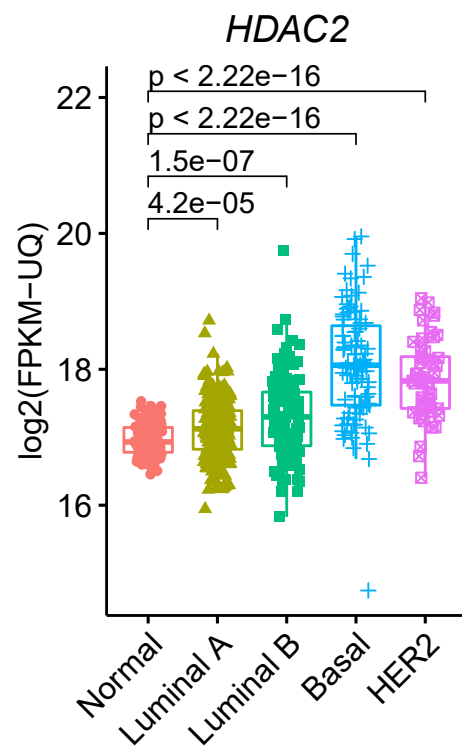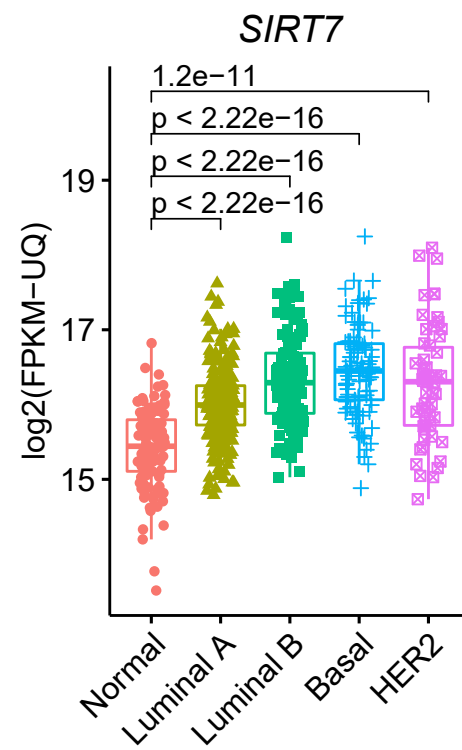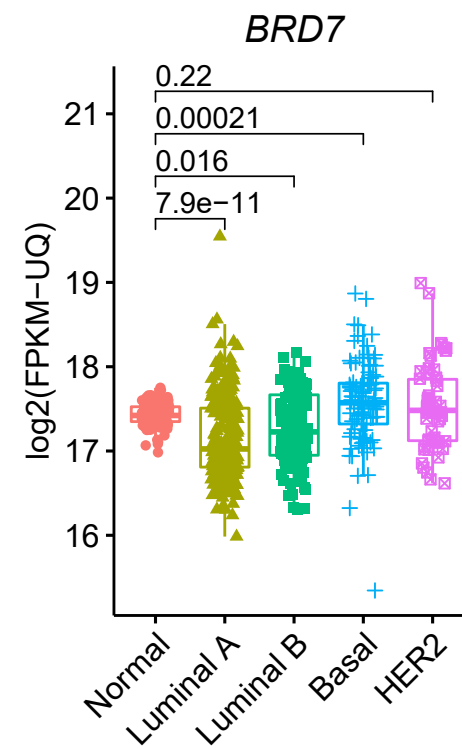

Supplement: Supplementary file 1 [file curroncol-28-00091-s001.zip › Figure S1.pdf]

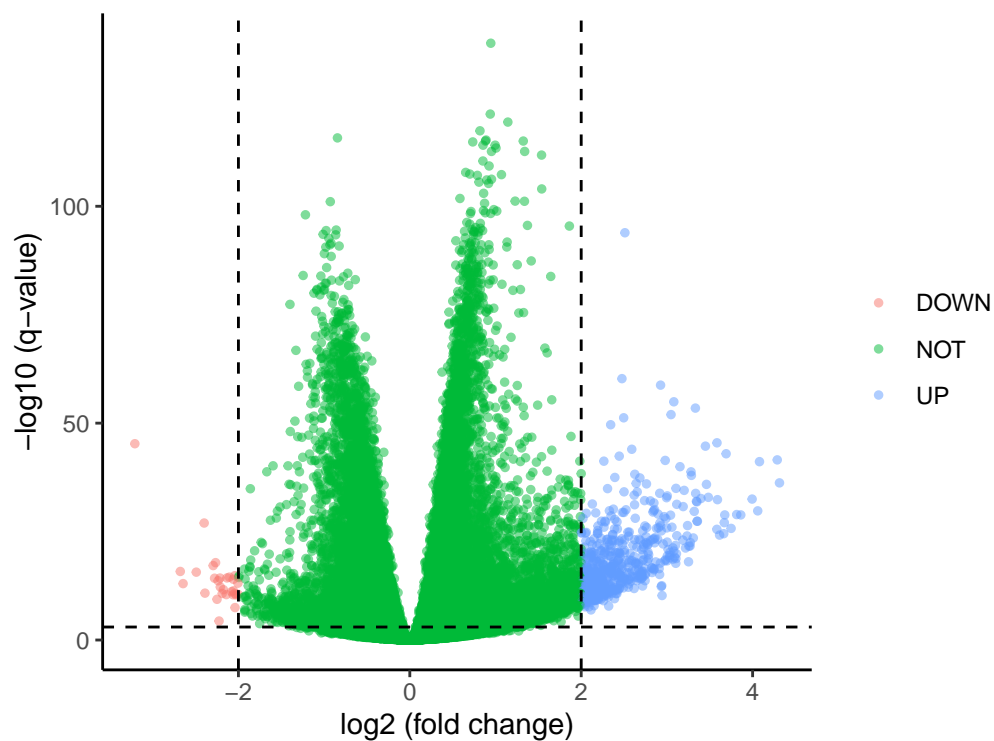

Supplement: Supplementary file 1 [file curroncol-28-00091-s001.zip › Figure S2.pdf]
